# Synthesis and Anticancer Activity Evaluation of Hydrolyzed Derivatives of *Panaxnotoginseng* Saponins

**DOI:** 10.3390/molecules23113021

**Published:** 2018-11-19

**Authors:** Lei Xu, Shengnan Xiao, Weihui Yuan, Jiongmo Cui, Guangyue Su, Yuqing Zhao

**Affiliations:** 1 School of Functional Food and Wine, Shenyang Pharmaceutical University, Shenyang 110016, China; 18246691730@163.com (L.X.); 15931605679@163.com (S.X.); 15142590591@163.com (W.Y.); 2 College of Pharmacy, Yanbian University, Yanji 133000, China; jmcui@ybu.edu.cn; 3 Key Laboratory of Structure-based Drug Design and Discovery of Ministry of Education, Shenyang Pharmaceutical University, Shenyang 110016, China

**Keywords:** ginsenoside, salicylic acid, acetylsalicylic acid, anti-cancer activity

## Abstract

To increase the antitumor activity of ginsenosides and acetylsalicylic acid, acid hydrolysis products of *Panaxnotoginseng* saponin were used as raw materials to be combined with salicylic acid to obtain ginsenoside salicylic acid derivatives. All derivatives were assessed for anti-cancer activity. A total of 20 target compounds were designed and synthesized. The cytotoxic activity on five cancer cell lines, including human colon cancer (HT-29), gastric cancer (BGC-823), cervical cancer (Hela), human breast cancer (MCF-7), human lung cancer cells (A549), and two normal cancer cell lines (human gastric epithelial cells (GES-1), and human ovarian epithelial cells (IOSE144)) was evaluated following treatment with the compounds. The results showed that all compounds inhibited the growth of cancer cells. Compounds **1a**, **3a**, **7a**, **1b**, **2b**, **3b** and **8b** showed strong anticancer activity. For MCF-7 cells, compound **3b** showed the strongest inhibitory activity, IC_50_ = 2.56 ± 0.09 μM. In the cytotoxicity test, all compounds showed low toxicity or no toxicity (IC_50_ > 100 μM). In addition, a cell cycle distribution assay and wound healing assay demonstrated that compound **3b** specifically inhibited MCF-7 proliferation and migration ability. Our results indicate that compound **3b** represents a promising compound for further cancer studies.

## 1. Introduction

**Figure 1:**
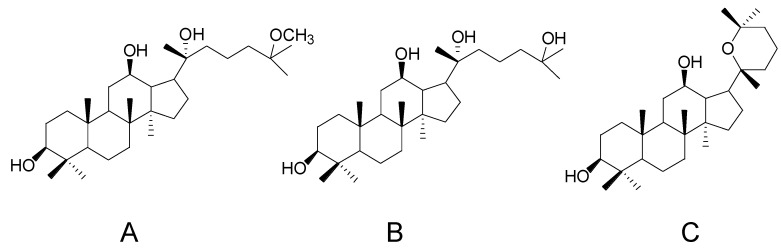
(**A**)The structure of 25-methoxyprotopanaxadiol (25-OCH_3_-PPD, AD-1); (**B**) the structureof 25-hydroxyprotopanaxadiol (25-OH-PPD, AD-2); (**C**) the structure of panaxadiol (PD).

*Panaxnotoginseng* is the ginseng plant of the genus Araliaceae. In modern scientific research, the main active ingredients of *Panaxnotoginseng* are ginsenosides [1]. Ginsenosides have been reported to have significant biological activities including anti-tumor activity [2,3,4,5], immune regulation [6], and significantly improve the quality of life [7]. For example, “Shenyi Capsule”, a new type of anti-cancer drug, was listed in 2003, and the main component of this drug is ginsenoside-Rg_3_. This provides an important opportunity for the full utilization and development of ginseng plants into medicinal resources. In our previous studies, we have proven that ginsenosides and their derivatives from ginseng, American ginseng, and *Panaxnotoginseng* have anti-tumor activity [8]. These include a series of studies on 25-methoxyprotopanaxadiol (25-OCH_3_-PPD, AD-1), 25-hydroxyprotopanaxadiol (25-OH-PPD, AD-2), panaxadiol (PD), and their homologs. The structures of AD-1, AD-2, and PD are shown in Figure 1. Although these compounds exhibit antitumor activity, the effect is not ideal, and whether this can be improved by additional chemical transformations is worth further investigation. Thus, new structure-activity relationships produced by changes to these compounds is a hot topic, including structural modifications of dammarane derivatives [9].

Dammarane compounds are abundantly present in ginseng plants. Accordingly, research and application of these components forcancer prevention therapies has become a research hotspot in international cancer studies. In our previous studies, AD-1 and AD-2 showed a 5–15-fold stronger antitumor activity when compared with ginsenoside-Rg_3_ [10]. Whilst performing structural modifications of lead compounds, combinations of AD-1, AD-2 with amino acids and fatty acids were synthesized. Cytotoxicity assays using cancer cell lines have demonstrated that the derivatives had anti-cancer activity, and had no effects on non-cancerous cells [11,12,13].

Acetylsalicylic acid is one of the world’s most used medicines. In recent years, it has been shown that acetylsalicylic acid also displays anti-tumor activity [14,15,16,17]. Moreover, it has been reported that acetylsalicylic acid platinum, the combination of acetylsalicylic acid with cisplatin, showed stronger anti-cancer activity than cisplatin or acetylsalicylic acid alone [18].

Salicylic acid is a precursor of acetylsalicylic acid. In recent years, new activities of salicylic acid, including anti-cardio cerebral vascular diseases, and anti-cancer activities have been described [19]. Liu and colleagues demonstrated synthesis of a salicylic acid derivative by the reaction of salicylic acid with α-aminophosphonate, and demonstrated its inhibitory activity against liver and cervical cancer cell lines [20].

Since both dammarane ginsenosides and salicylic acid have antitumor activity we hypothesized that the combination of antitumor compounds with acetylsalicylic acid may enhance the anticancer activity of specific substrates.

Whether the combination of dammarane ginsenosides and salicylic acid can produce better anti-tumor activity is not presently known. In this study, we combined acid hydrolysates derived from *Panaxnotoginseng* saponins with salicylic acid and acetylsalicylic acid. In addition, we identified a molecule with stronger anti-tumor activity and potential research value through testing the cytotoxicity on five cancer cell lines.

## 2. Results

A total of 20 compounds were synthesized, including 14 ginsenoside salicylic acid derivatives and six ginsenoside acetylsalicylic acid derivatives. Their structures are shown in Table 1.

### 2.1. Cytotoxicity Assay

Cytotoxicity results are summarized in Table 2. Compounds **1a**, **3a**, **7a**, **1b**, **2b**, **3b** and **8b** showed strong anticancer activity. For MCF-7 cells, compound **3b** showed the strongest inhibitory activity, IC_50_ = 2.56 ± 0.09 μM. In the cytotoxicity tests, all compounds showed low toxicity or no toxicity (IC_50_ > 100 μM).

### 2.2. Cell Cycle Distribution Assay

**Figure 2:**
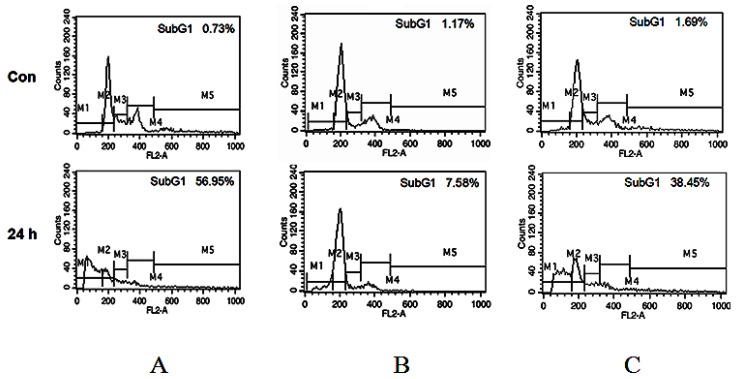
Cell cycle distribution assay. (**A**) MCF-7 cells treated with compound **3b**; (**B**) IOSE144 cells treated with compound **3b**; (**C**) MCF-7 cells treated with positive control (AD-1).

As compound **3b** had significant anti-proliferative effects on MCF-7 cells, cell cycle analysis was performed. The results indicated that the subG1 phase increased from 0.73% to 56.95% after treatment with compound **3b**. The effects on IOSE144 cells showed that the subG1 phase increased from 1.17% to 7.58% after treatment with compound **3b**. In addition, the positive control (AD-1) showed that subG1 phase of MCF-7 cells increased from 1.69% to 38.45% after treatment with AD-1 (Figure 2).

### 2.3. Wound Healing Assay

**Figure 3:**
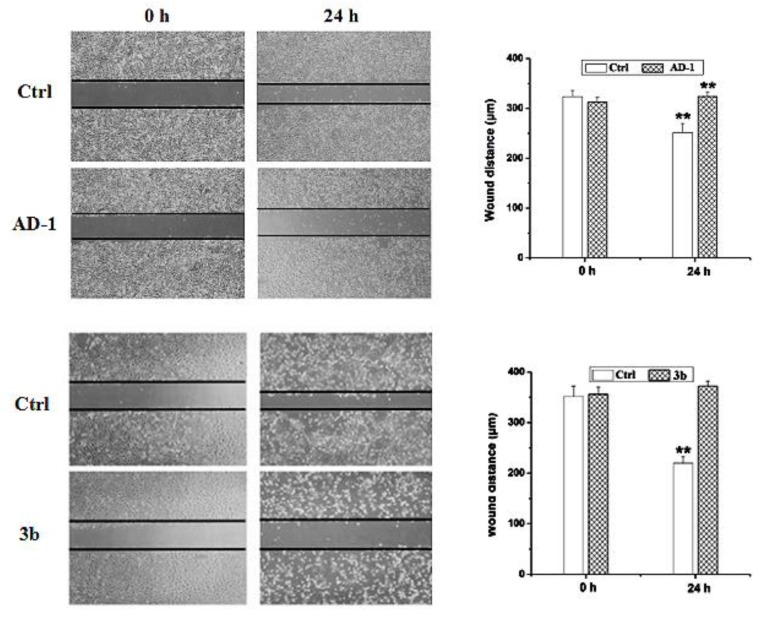
Wound distance of MCF-7 with **3b** and AD-1.

In our study, the wound healing assays showed that the distance of MCF-7 cell migration after treatment with compound **3b** was almost unchanged, whereas the healing distance of MCF-7 cells in the control group gradually decreased. The results of the positive control indicated that AD-1 inhibited the migration of MCF-7 cells, which was comparable with the results of compound **3b** (Figure 3).

## 3. Discussion

In this study, we designed and synthesized 20 ginsenoside derivatives, including ginsenoside salicylic acid derivatives and ginsenoside acetylsalicylic acid derivatives. Through evaluation of their anticancer activity, we found that the anticancer activity of ginsenoside acetylsalicylic acid derivatives was significantly stronger when compared to that of ginsenoside salicylic acid derivatives. Moreover, we also obtained a series of more active compounds (compounds **1a**, **3a**, **7a**, **1b**, **2b**, **3b**, and **8b**). Since compound **3b** was found to have the strongest anticancer activity against MCF-7 cells (IC_50_ = 2.56 ± 0.09 μM), we conducted further studies, including cell cycle distribution assay and wound healing assay, and obtained promising results that provide theoretical and data support for further studies. In conclusion, this study highlights the potential to develop novel and potent compounds for cancer prevention and therapy.

## 4. Materials and Methods

### 4.1. Synthesis of Salicylic Acid Derivatives

**Scheme 1:**
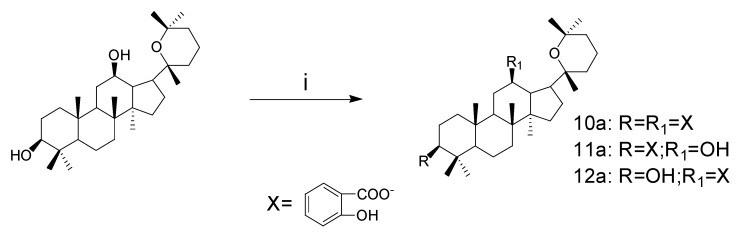
Synthesis of compound **10a**–**12a**. i. DCC, DMAP, Salicylic acid, CH_2_Cl_2_, r.t, 24h.

**Scheme 2:**
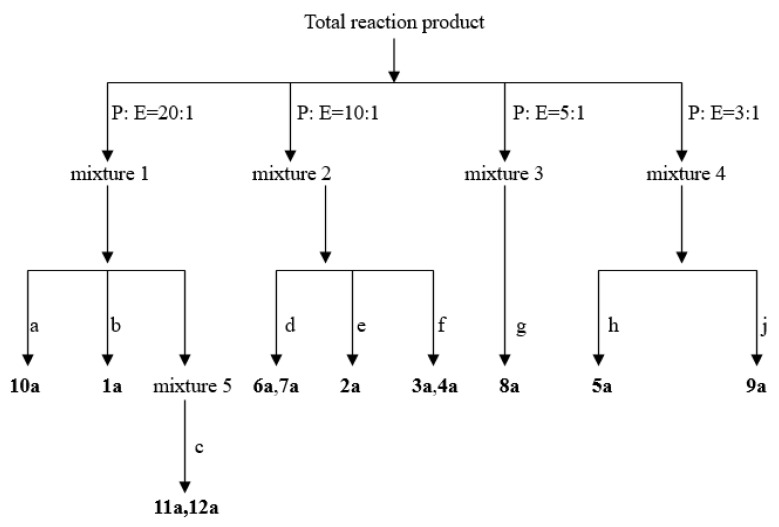
Separation process of ginsenoside salicylic acid derivatives. a: P:E = 25:1; b: P:E = 20:1; c: P:A = 6:1 (Thin layer chromatography); d: P:A = 4:1 (Thin layer chromatography); e: P:E = 10:1; f: P:E = 8:1; g: P:E = 6:1; h: P:A = 6:1; j: P:E = 1:1. P: Petroleum ether; E: Ethyl acetate; A: Acetone.

The ginsenoside salicylic acid derivative was synthesized as follows: the acid hydrolyzate of *Panaxnotoginseng* saponin (1 equiv, 5 g, 11 mmol) was dissolved in dry dichloromethane, and stirred for 20 min. Then, *N*,*N*’-dicyclohexylcarbodiimide (DCC, 2 equiv, 4.5 g), salicylic acid (2 equiv, 3 g), and 4-dimethylaminopyridine (DMAP, 1 equiv, 1.3 g) were added in sequence, and reacted at room temperature for 24 h [21] (Scheme 1). The total reaction product was separated by a silica gel column (Scheme 2). We will use the panaxadiol in the hydrolysate of *Panaxnotoginseng* saponin as an example to illustrate the reaction process.

### 4.2. Synthesis of Acetylsalicylic Acid Derivatives

**Scheme 3:**
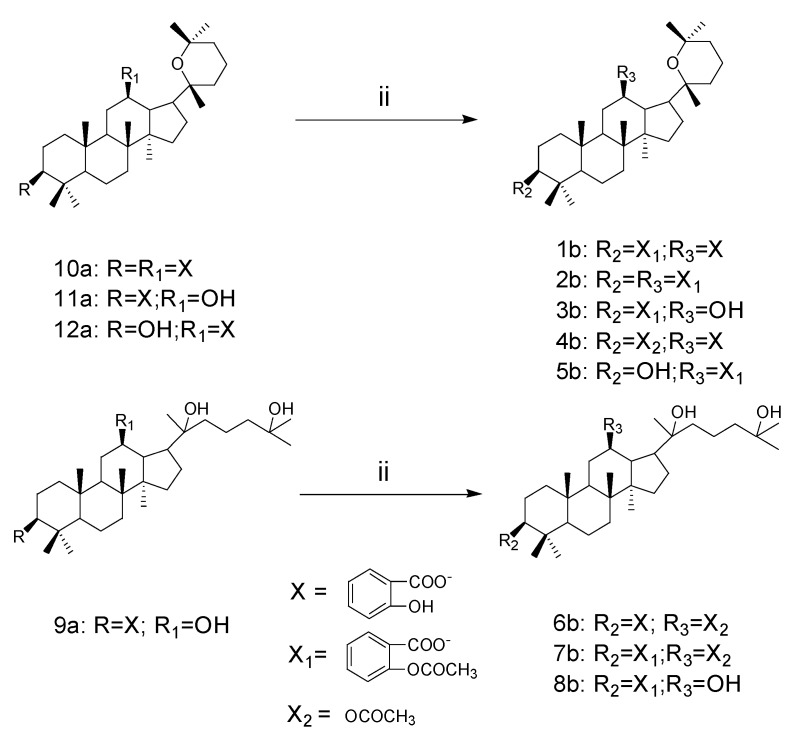
Synthesis of compound **1b**–**8b**. ii: Acetic anhydride, Triethylamine, CH_2_Cl_2_, r.t, 2h.

A total of 120 mg, 100 mg, 200 mg, and 100 mg of compound **9a**, **10a**, **11a**, and **12a** were dissolved in 10 mL of dry dichloromethane solution, respectively, and 10 drops of triethylamine (C_6_H_15_N) were added in a dropwise fashion to provide an alkaline environment. The molar ratio of the reaction substrate was 1:2. Next, excess acetic anhydride was added (0.5 mL) and the reaction was carried out for 2 h at room temperature. All compounds were isolated by repeated column chromatography (Scheme 3).

A total of 20 ginsenoside salicylic acid derivatives and ginsenoside acetylsalicylic acid derivatives were obtained (Table 1).

### 4.3. Structure Identification of Compounds

Melting points were determined in open capillary tubes. The ^1^H-NMR and ^13^C-NMR spectra were measured on an AV-400 spectrometer (Bruker, Karlsruhe, Germany) in CDCl_3_ solutions using tetramethylsilane as the internal standard. Mass spectra were obtained on a Quattro Micromass instrument (Waters, Milford, MA, USA). As shown in our previous work, the hydroxyl groups of 25-OCH_3_–PPD, 25-OH-PPD or PD were replaced. In the ^13^C-NMR spectrum, the signals changed from δC 78.0–79.0 (C-3), 70.5–71.5 (C-12) to δC 81.0–83.0 (C-3), 75.0–77.0 (C-12), respectively. In the ^1^H-NMR spectrum, signals changed from δH 3.2 (H-3), 3.6 (H-12) to δH 4.7–4.8 (H-3), 5.1–5.2 (H-12), respectively. Combining the spectrum of salicylic acid and acetylsalicylic acid, we could accurately determine the structures of the derivatives on the basis of 25-OH-PPD, 25-OCH_3_-PPD or PD. The spectrums (see Table 1) of all compounds are in Supplementary Materials.

*20(R)-Dammarane-20-hydroxy-3β,12β,25-tri-yl-2′-hydroxybenzoate* (**1a**, C_51_H_66_O_10_). White solid, melting point: 192–194 °C, ^1^H-NMR (CDCl_3_, 400 MHz, ppm): δ 0.94 (s, 3H), 0.95 (s, 3H), 1.02 (s, 3H), 1.09 (s, 3H), 1.59 (s, 6H), 1.62 (s, 6H), 4.77 (td, *J*_1,3_ = 8.0 Hz, *J*_1,2_ = 4.0 Hz, 1H, H-3), 5.20 (td, *J*_1,3_ = 12.0 Hz, *J*_1,2_ = 8.0 Hz, 1H, H-12), 6.82–6.89 (m, 3H, Ar-H), 6.97 (t, *J*_1,2_ = 8 Hz, *J*_1,3_ = 20.0 Hz, 3H, Ar-H), 7.44 (td, *J*_1,3_ = 8.0 Hz, *J*_1,2_ = 8.0 Hz, 3H, Ar-H), 7.81 (dd, *J*_1,3_ = 28.0 Hz, *J*_1,2_ = 8.0 Hz, 3H, Ar-H), 10.79 (s, 1H, AR-OH), 10.92 (s, 1H, Ar-OH), 11.04 (s, 1H, Ar-OH); ^13^C-NMR (CDCl_3_, 100 MHz, ppm): 39.85 (C-1), 26.44 (C-2), 82.04 (C-3), 38.40 (C-4), 56.05 (C-5), 18.16 (C-6), 34.55 (C-7), 38.61 (C-8), 51.75 (C-9), 37.25 (C-10), 33.20 (C-11), 77.37 (C-12), 49.94 (C-13), 53.46 (C-14), 31.11 (C-15), 26.36 (C-16), 52.91(C-17), 16.87 (C-18), 15.82 (C-19), 73.93 (C-20), 23.69 (C-21), 41.87 (C-22), 18.30 (C-23), 44.97 (C-24), 85.34 (C-25), 28.48 (C-26), 28.30 (C-27), 26.91 (C-28), 16.28 (C-29), 17.28 (C-30). Structure of salicylic acid: 112.71, 113.14, 113.93 (C-1), 117.66, 117.74, 118.07 (C-3), 119.08, 119.18, 119.32 (C-5), 129.36, 129.85, 130.23 (C-6), 135.37, 135.63, 136.02 (C-4), 161.88, 161.91, 162.13 (C-2), 169.40, 169.94, 170.04 (–C=O). MS: *m*/*z* 838.47 [M + H]^+^.

*20(R)-25-Methoxydammarane-20-hydroxy-3β,12β-di-yl-2′-hydroxybenzoate* (**2a**, C_45_H_64_O_8_). White solid, melting point: 200–202 °C, ^1^H-NMR (CDCl_3_, 400 MHz, ppm): δ 0.94 (s, 6H), 1.02 (s, 3H), 1.03 (s, 3H), 1.08 (s, 3H), 1.09 (s, 3H), 1.14 (s, 3H), 1.16 (s, 3H), 4.75–4.79 (dd, *J*_1,3_ = 12.0 Hz, 11H, H-3), 5.18–5.25 (td, *J*_1,3_ = 12.0 Hz, *J*_1,2_ = 8.0 Hz, 1H, H-12), 6.84–6.88 (td, *J*_1,3_ = 12.0 Hz, *J*_1,2_ = 4.0 Hz, 2H, Ar-H), 6.94–6.98 (t, *J*_1,3_ = 16.0 Hz, *J*_1,2_ = 8.0 Hz, 2H, Ar-H), 7.41–7.46 (m, 2H, Ar-H), 7.73–7.82 (m, 2H, Ar-H), 10.82 (s, 1H, Ar-OH), 10.92 (s, 1H, Ar-OH); ^13^C-NMR (CDCl_3_, 100 MHz, ppm): 38.57 (C-1), 28.25 (C-2), 82.01 (C-3), 39.82 (C-4), 56.00 (C-5), 18.24 (C-6), 34.51 (C-7), 38.30 (C-8), 49.90 (C-9), 37.22 (C-10), 31.08 (C-11), 77.28 (C-12), 49.26 (C-13), 53.27 (C-14), 30.75 (C-15), 26.44 (C-16), 52.35 (C-17), 16.24 (C-18), 16.85 (C-19), 73.99 (C-20), 23.66 (C-21), 44.96 (C-22), 18.01 (C-23), 41.13 (C-24), 74.73 (C-25), 25.21 (C-26), 24.91 (C-27), 28.45 (C-28), 15.78 (C-29), 17.26 (C-30). Structure of salicylic acid: 112.78, 113.10 (C-1), 117.70, 117.90 (C-3), 119.14, 119.25 (C-5), 129.42, 129.81 (C-6), 135.59, 135.87 (C-4), 161.85, 162.11 (C-2), 169.38, 169.90 (–C=O); MS: *m*/*z* 731.91 [M − H]^+^.

*20(R)-25-Methoxydammarane-3β,20-diol-12β-yl-2′-hydroxybenzoate* (**3a**, C_38_H_60_O_6_). White solid, melting point: 235–237 °C, ^1^H-NMR (CDCl_3_, 400 MHz, ppm): δ 0.75 (s, 3H), 0.83 (s, 3H), 0.95 (s, 3H), 0.99 (s, 3H), 1.04 (s, 3H), 1.07 (s, 3H), 1.12 (s, 3H), 1.13 (s, 3H), 3.19 (dd, *J*_1,2_ = 4.0 Hz, *J*_1,3_ = 8.0 Hz, 1H, H-3), 5.17 (td, *J*_1,3_ = 12.0 Hz, *J*_1,2_ = 4.0 Hz, 1H, H-12), 6.84 (t, *J*_1,3_ = 12.0 Hz, *J*_1,2_ = 4.0 Hz, 1H, Ar-H), 6.94 (d, *J*_1,2_ = 8.0 Hz, 1H, Ar-H), 7.41 (td, *J*_1,3_ = 8.0 Hz, *J*_1,2_ = 4.0 Hz, 1H, Ar-H), 7.73 (dd, *J*_1,3_ = 8.0 Hz, *J*_1,2_ = 4.0 Hz, 1H, Ar-H), 10.81 (d, 1H, Ar-OH); ^13^C-NMR (CDCl_3_, 100 MHz, ppm): 38.96 (C-1), 27.25 (C-2), 78.65 (C-3), 39.70 (C-4), 55.82 (C-5), 18.27 (C-6), 34.54 (C-7), 38.85 (C-8), 49.92 (C-9), 37.18 (C-10), 31.17 (C-11), 77.25 (C-12), 49.18 (C-13), 52.80 (C-14), 31.00 (C-15), 26.33 (C-16), 50.00 (C-17), 15.69 (C-18), 16.12 (C-19), 73.95 (C-20), 23.03 (C-21), 44.88 (C-22), 17.93 (C-23), 41.00 (C-24), 74.70 (C-25), 25.15 (C-26), 24.87 (C-27), 28.07 (C-28), 15.44 (C-29), 17.19 (C-30), 49.09 (–OCH_3_). Structure of salicylic acid: 112.72 (C-1), 117.90 (C-3), 119.22 (C-5), 129.39 (C-6), 135.82 (C-4), 161.98 (C-2), 169.39 (–C=O).

*20(R)-25-Methoxydammarane-12β,20-diol-3β-yl-2′-hydroxybenzoate* (**4a**, C_38_H_60_O_6_). White solid, melting point: 246–248 °C, ^1^H-NMR (CDCl_3_, 400 MHz, ppm): δ 0.91 (s, 3H), 0.94 (s, 3H), 0.96 (s, 3H), 1.01 (s, 3H), 1.02 (s, 3H), 1.16 (s, 6H), 1.21 (s, 3H), 3.59–3.65 (td, *J*_1,3_ = 8.0 Hz, *J*_1,2_ = 4.0 Hz, 1H, H-12), 4.74–4.78 (dd, *J*_1,3_ = 12.0 Hz, *J*_1,2_ = 8.0 Hz, 1H, H-3), 6.85–6.90 (t, *J*_1,3_ = 16.0 Hz, *J*_1,2_ = 6.0 Hz, 1H, Ar-H), 6.97–6.99 (d, *J*_1,2_ = 8.0 Hz, 1H, Ar-H), 7.43–7.47 (td, *J*_1,3_ = 8.0 Hz, *J*_1,2_ = 3.0 Hz, 1H, Ar-H), 7.82–7.84 (dd, *J*_1,3_ = 8.0 Hz, *J*_1,2_ = 3.0 Hz, 1H, Ar-H), 10.93 (s, 1H, Ar-OH); ^13^C-NMR (CDCl_3_, 100 MHz, ppm): 38.72 (C-1), 27.24 (C-2), 82.32 (C-3), 39.92 (C-4), 56.12 (C-5), 18.31 (C-6), 34.82 (C-7), 38.38 (C-8), 50.09 (C-9), 37.20 (C-10), 31.30 (C-11), 70.95 (C-12), 49.29 (C-13), 53.61 (C-14), 31.13 (C-15), 26.63 (C-16), 51.72 (C-17), 16.33 (C-18), 16.87 (C-19), 74.63 (C-20), 23.82 (C-21), 42.82 (C-22), 17.88 (C-23), 40.68 (C-24), 74.87 (C-25), 25.25 (C-26), 25.12 (C-27), 28.29 (C-28), 15.80 (C-29), 16.97 (C-30), 47.95 (OCH_3_). Structure of salicylic acid: 113.17 (C-1), 117.71 (C-3), 119.20 (C-5), 129.89 (C-6), 135.61 (C-4), 161.85 (C-2), 170.03 (–C=O). MS: *m*/*z* 612.88 [M + H]^+^.

*20(R)-Dammarane-3β,20,25-triol-12β-yl-2′-hydroxybenzoate* (**5a**, C_37_H_58_O_6_). White solid, melting point: 258–260 °C, ^1^H-NMR (CDCl_3_, 400 MHz, ppm): δ 0.77 (s, 3H), 0.85 (s, 3H), 0.97 (s, 3H), 1.02 (s, 6H), 1.06 (s, 3H), 1.15, (s, 6H), 1.18 (s, 3H), 3.18–3.22 (dd, *J*_1,3_ = 12.0 Hz, *J*_1,2_ = 4.0 Hz, 1H, H-3), 5.16–5.22 (td, *J*_1,3_ = 8.0 Hz, *J*_1,2_ = 4.0 Hz, 1H, H-12), 6.87–6.91 (t, *J*_1,3_ = 16.0 Hz, *J*_1,2_ = 8.0 Hz, 1H, Ar-H), 6.94–6.96 (d, *J*_1,2_ = 8.0 Hz, 1H, Ar-H), 7.41–7.45 (td, *J*_1,3_ = 8.0 Hz, *J*_1,2_ = 2.8 Hz, 1H, Ar-H), 7.76–7.79 (dd, *J*_1,3_ = 8.0 Hz, *J*_1,2_ = 4.0 Hz, 1H, Ar-H), 10.83 (s, 1H, Ar-OH); ^13^C-NMR (CDCl_3_, 100 MHz, ppm): 39.68 (C-1), 28.00 (C-2), 78.68 (C-3), 38.90 (C-4), 55.78 (C-5), 17.76 (C-6), 37.14 (C-7), 42.64 (C-8), 50.00 (C-9), 38.80 (C-10), 33.58 (C-11), 77.48 (C-12), 49.45 (C-13), 53.47 (C-14), 31.19 (C-15), 27.09 (C-16), 52.84 (C-17), 15.62 (C-18), 16.10 (C-19), 74.01 (C-20), 23.00 (C-21), 44.26 (C-22), 18.22 (C-23), 45.43 (C-24), 71.26 (C-25), 29.28 (C-26), 29.02 (C-27), 28.38 (C-28), 15.39 (C-29), 17.59 (C-30). Structure of salicylic acid: 112.63 (C-1), 117.83 (C-3), 119.47 (C-5), 129.37 (C-6), 135.87 (C-4), 161.90 (C-2), 169.42 (–C=O). MS: *m*/*z* 598.85 [M + H]^+^.

*20(R)-Dammarane-12β, 20-diol-3β, 25-di-yl-2′-hydroxybenzoate* (**6a**, C_44_H_62_O_8_). White solid, melting point: 219–221 °C, ^1^H-NMR (CDCl_3_, 400 MHz, ppm): δ 0.84 (s, 3H), 0.93 (s, 3H), 0.95 (s, 3H), 1.00 (s, 3H), 1.02 (s, 3H), 1.14 (s, 3H), 1.60 (s, 3H), 1.62 (s, 3H), 3.59–3.66 (td, *J*_1,3_ = 12.0 Hz, 1H, H-12), 4.73–4.77 (dd, *J*_1,3_ = 12.0 Hz, *J*_1,2_ = 8.0 Hz, 1H, H-3), 6.82–6.89 (m, 2H, Ar-H), 6.94–6.99 (m, 2H, Ar-H), 7.39–7.47 (m, 2H, Ar-H), 7.76–7.84 (m, 2H, Ar-H), 10.92 (s, 1H, Ar-OH), 11.02 (s, 1H, Ar-OH); ^13^C-NMR (CDCl_3_, 100 MHz, ppm): 39.91 (C-1), 26.51 (C-2), 82.29 (C-3), 38.40 (C-4), 56.12 (C-5), 18.31 (C-6), 34.80 (C-7), 41.61 (C-8), 51.02 (C-9), 37.20 (C-10), 31.57 (C-11), 71.19 (C-12), 49.96 (C-13), 53.56 (C-14), 31.09 (C-15), 26.31 (C-16), 51.80 (C-17), 16.34 (C-18), 16.87 (C-19), 74.31 (C-20), 23.81 (C-21), 43.00 (C-22), 22.33 (C-23), 48.48 (C-24), 85.28 (C-25), 28.77 (C-26), 28.30 (C-27), 27.73 (C-28), 15.82 (C-29), 17.68 (C-30). Structure of salicylic acid: 113.18, 113.93 (C-1), 117.68, 117.74 (C-3), 119.05, 119.22 (C-5), 129.90, 130.20 (C-6), 135.35, 135.64 (C-4), 161.87, 161.93 (C-2), 169.93, 170.05 (–C=O). MS: *m*/*z* 718.96 [M + H]^+^.

*20(R)-Dammarane-20, 25-diol-3β, 12β-di-yl-2′-hydroxybenzoate* (**7a**, C_44_H_62_O_8_). White solid, melting point: 197–199 °C, ^1^H-NMR (CDCl_3_, 400 MHz, ppm): δ 0.95 (s, 6H), 1.02 (s, 3H), 1.05 (s, 3H), 1.09 (s, 3H), 1.17 (s, 6H), 1.19 (s, 3H), 4.76–4.80 (dd, *J*_1,3_ = 12.0 Hz, *J*_1,2_ = 4.0 Hz, 1H, H-3), 5.19–5.25 (td, *J*_1,3_ = 8.0 Hz, *J*_1,3_ = 4.0 Hz, 1H, H-12), 6.85–6.92 (m, 2H, Ar-H), 6.96–6.99 (m, 2H, Ar-H), 7.42–7.46 (m, 2H, Ar-H), 7.77–7.83 (m, 2H, Ar-H), 10.92 (s, 1H, Ar-OH); ^13^C-NMR (CDCl_3_, 100 MHz, ppm): 38.61 (C-1), 28.28 (C-2), 82.03 (C-3), 38.39 (C-4), 56.06 (C-5), 17.93 (C-6), 34.55 (C-7), 39.87 (C-8), 49.60 (C-9), 37.26 (C-10), 33.80 (C-11), 77.25 (C-12), 49.53 (C-13), 52.96 (C-14), 31.35 (C-15), 26.97 (C-16), 50.06 (C-17), 16.30 (C-18), 16.86 (C-19), 73.95 (C-20), 23.16 (C-21), 44.47 (C-22), 18.28 (C-23), 45.59 (C-24), 71.23 (C-25), 29.52 (C-26), 28.25 (C-27), 28.57 (C-28), 15.79 (C-29), 17.75 (C-30). Structure of salicylic acid: 112.75, 113.13 (C-1), 117.73, 118.00 (C-3), 119.17, 119.58 (C-5), 129.45, 129.83 (C-6), 135.62, 136.00 (C-4), 161.85, 162.09 (C-2), 169.52, 169.93 (–C=O).

*20(R)-Dammarane-3β,12β,20-triol-25-yl-2′-hydroxybenzoate* (**8a**, C_37_H_58_O_6_). White solid, melting point: 223–225 °C, ^1^H-NMR (CDCl_3_, 400 MHz, ppm): δ 0.74 (s, 3H), 0.79 (s, 3H), 0.82 (s, 3H), 0.94 (s, 6H), 1.08 (s, 3H), 1.56 (s, 3H), 1.58 (s, 3H), 3.14–3.18 (dd, *J*_1,3_ = 12.0 Hz, *J*_1,2_ = 4.0 Hz, 1H, H-3), 3.52–3.58 (td, *J*_1,3_ = 12.0 Hz, *J*_1,2_ = 4.0 Hz, 1H, H-12), 6.78–6.82 (t, *J*_1,3_ = 16.0 Hz, *J*_1,2_ = 8.0 Hz, 1H, Ar-H), 6.91–6.93 (d, *J* = 8.0 Hz, 1H, Ar-H), 7.36–7.43 (m, 1H, Ar-H), 8.00–8.07 (dd, *J*_1,3_ = 20.0 Hz, *J*_1,2_ = 8.0 Hz, 1H, Ar-H), 11.03 (s, 1H, Ar-OH); ^13^C-NMR (CDCl_3_, 100 MHz, ppm): 39.70 (C-1), 26.08 (C-2), 78.88 (C-3), 38.93 (C-4), 55.84 (C-5), 17.56 (C-6), 34.76 (C-7), 41.60 (C-8), 50.08 (C-9), 37.06 (C-10), 33.62 (C-11), 71.04 (C-12), 49.65 (C-13), 53.52 (C-14), 31.19 (C-15), 24.87 (C-16), 51.59 (C-17), 15.63 (C-18), 16.18 (C-19), 74.14 (C-20), 22.00 (C-21), 42.64 (C-22), 18.28 (C-23), 48.22 (C-24), 85.27 (C-25), 28.05 (C-26), 27.24 (C-27), 26.33 (C-28), 15.45 (C-29), 17.03 (C-30). Structure of salicylic acid: 113.84 (C-1), 117.52 (C-3), 118.07 (C-5), 130.12 (C-6), 135.22 (C-4), 161.74 (C-2), 169.83 (–C=O). MS: *m*/*z* 597.45 [M − H]^+^.

*20(R)-Dammarane-12β,20,25-triol-3β-yl-2′-hydroxybenzoate* (**9a**, C_37_H_58_O_6_). White solid, melting point: 295–297 °C, ^1^H-NMR (CDCl_3_, 400 MHz, ppm): δ 0.91 (s, 3H), 0.94 (s, 3H), 0.96 (s, 3H), 1.01 (s, 3H), 1.02 (s, 3H), 1.14 (s, 3H), 1.24 (s, 6H), 3.63 (td, *J*_1,3_ = 10.2 Hz, *J*_1,2_ = 5.36 Hz, 1H, H-12), 4.75 (dd, *J*_1,3_ = 10.2 Hz, *J*_1,2_ = 5.88 Hz, 1H, H-3), 6.88 (t, *J*_1,3_ = 14.84 Hz, *J*_1,2_ = 7.4 Hz, 1H, Ar-H), 6.99 (d, *J* = 8.28 Hz, 1H, Ar-H), 7.44 (td, *J*_1,3_ = 8.52 Hz, *J*_1,2_ = 1.48 Hz, 1H, Ar-H), 7.83 (dd, *J*_1,3_ = 7.6 Hz, *J*_1,2_ = 1.52 Hz, 1H, Ar-H), 10.93 (s, 1H, Ar-OH); ^13^C-NMR (CDCl_3_, 100 MHz, ppm): 38.74 (C-1), 28.30 (C-2), 82.32 (C-3), 38.39 (C-4), 56.13 (C-5), 17.85 (C-6), 34.82 (C-7), 39.92 (C-8), 50.11 (C-9), 37.21 (C-10), 31.45 (C-11), 71.29 (C-12), 48.65 (C-13), 53.57 (C-14), 31.16 (C-15), 36.52 (C-16), 51.78 (C-17), 16.35 (C-18), 16.88 (C-19), 74.57 (C-20), 22.04 (C-21), 43.12 (C-22), 18.32 (C-23), 44.28 (C-24), 71.00 (C-25), 29.84 (C-26), 29.59 (C-27), 29.55 (C-28), 15.81 (C-29), 17.30 (C-30). Structure of salicylic acid: 113.18 (C-1), 117.72 (C-3), 119.91 (C-5), 129.91 (C-6), 135.63 (C-4), 161.87 (C-2), 170.04 (–C=O).

*20(R)-Pananxadiol-3β,12β-di-yl-2′-hydroxybenzoate* (**10a**, C_44_H_60_O_7_). White solid, melting point: 232–234 °C, ^1^H-NMR (CDCl_3_, 400 MHz, ppm): δ 0.65 (s, 3H), 0.91 (s, 3H), 0.92 (s, 3H), 0.94 (s, 3H), 1.00 (s, 3H), 1.01 (s, 3H), 1.05 (s, 3H), 1.16 (s, 3H), 4.75–4.85 (m, *2*H, H-12, H-3), 6.85–6.89 (t, *J*_1,3_ = 15.36 Hz, *J*_1,2_ = 8.62 Hz, 2H, Ar-OH), 6.95–6.98 (d, *J* = 12.0 Hz, 2H, Ar-H), 7.64–7.69 (td, *J*_1,2_ = 4.0 Hz, *J*_1,3_ = 8.0Hz, 2H, Ar-H), 7.98–8.08 (m, *2*H, Ar-H), 10.92 (s, 1H, Ar-OH), 11.19 (s, 1H, Ar-OH); ^13^C-NMR (CDCl_3_, 100 MHz, ppm): 38.52 (C-1), 28.31 (C-2), 82.14 (C-3), 38.39 (C-4), 55.96 (C-5), 18.31 (C-6), 34.29 (C-7), 39.69 (C-8), 49.55 (C-9), 37.37 (C-10), 30.44 (C-11), 75.70 (C-12), 49.10 (C-13), 51.92 (C-14), 31.42 (C-15), 25.24 (C-16), 54.35 (C-17), 15.96 (C-18), 16.73 (C-19), 75.03 (C-20), 23.71 (C-21), 35.37 (C-22), 16.90 (C-23), 37.16 (C-24), 70.80 (C-25), 33.05 (C-26), 26.80 (C-27), 28.45 (C-28), 15.91 (C-29), 18.02 (C-30). Structure of salicylic acid: 113.16, 114.04 (C-1), 116.28, 117.72 (C-3), 118.94, 119.16 (C-5), 129.85, 130.30 (C-6), 135.59, 135.97 (C-4), 161.23, 161.87 (C-2), 169.78, 169.95 (C-7, –C=O); MS: *m*/*z* 700.94 [M + H]^+^.

*20(R)-Pananxadiol-12β-hydroxy-3β-yl-2′-hydroxybenzoate* (**11a**, C_37_H_56_O_5_). White solid, melting point: 222–224 °C, ^1^H-NMR (CDCl_3_, 400 MHz, ppm): δ 0.90 (s, 3H), 0.93 (s, 3H), 0.95 (s, 3H), 1.00 (s, 3H), 1.01 (s, 3H), 1.19 (s, 3H), 1.22 (s, 3H), 1.27 (s, 3H), 3.52–3.58 (td, *J*_1,3_ = 8.0 Hz, *J*_1,2_ = 4.0 Hz, 1H, H-12), 4.73–4.77 (dd, *J*_1,3_ = 8.0 Hz, *J*_1,2_ = 4.0 Hz, 1H, H-3), 6.85–6.89 (t, *J*_1,3_ = 16.0 Hz, *J*_1,2_ = 8.0 Hz, 1H, Ar-H), 6.89–6.96 (d, *J*_1,2_ = 12.0 Hz, 1H, Ar-H), 7.42–7.46 (t, *J*_1,3_ = 16.0 Hz, *J*_1,2_ = 8.0 Hz, 1H, Ar-H), 7.82–7.84 (d, *J*_1,2_ = 8.0 Hz, 1H, Ar-H), 10.93 (s, 1H, Ar-OH); ^13^C-NMR (CDCl_3_, 100 MHz, ppm): 38.70 (C-1), 27.29 (C-2), 82.45 (C-3), 39.39 (C-4), 56.17 (C-5), 18.36 (C-6), 34.94 (C-7), 39.98 (C-8), 50.00 (C-9), 37.24 (C-10), 30.72 (C-11), 70.02 (C-12), 49.31 (C-13), 51.30 (C-14), 31.28 (C-15), 23.89 (C-16), 54.87 (C-17), 16.32 (C-18), 16.42 (C-19), 76.67 (C-20), 19.57 (C-21), 35.89 (C-22), 16.85 (C-23), 36.59 (C-24), 73.27 (C-25), 33.17 (C-26), 25.31 (C-27), 28.30 (C-28), 15.80 (C-29), 17.20 (C-30). Structure of salicylic acid: 113.22 (C-1), 117.70 (C-3), 119.18 (C-5), 129.91 (C-6), 135.57 (C-4), 161.87 (C-2), 170.05 (–C=O); MS: *m*/*z* 603.42 [M + Na]^+^.

*20(R)-Pananxadiol-3β-hydroxy-12β-yl-2′-hydroxybenzoate* (**12a**, C_37_H_58_O_6_). White solid, melting point: 248–250 °C, ^1^H-NMR (CDCl_3_, 400 MHz, ppm): δ 0.64 (s, 3H), 0.77 (s, 3H), 0.84 (s, 3H), 0.90 (s, 3H), 0.98 (s, 6H), 1.03 (s, 3H), 1.15 (s, 3H), 3.18–3.22 (dd, *J*_1,3_ = 8.0 Hz, *J*_1,2_ = 4.0 Hz, 1H, H-3), 6.85–6.89 (t, *J*_1,3_ = 16.0 Hz, *J*_1,2_ = 8.0 Hz, 1H, Ar-H), 6.95–6.98 (d, *J*_1,2_ = 12.0 Hz, 1H, Ar-H), 7.40–7.45 (td, *J*_1,2_ = 4.0 Hz, *J*_1,3_ = 8.0 Hz, 1H, Ar-H), 7.85–7.88 (dd, *J*_1,3_ = 8.0 Hz, *J*_1,2_ = 4.0 Hz, 1H, Ar-H), 11.20 (s, 1H, Ar-OH); ^13^C-NMR (CDCl_3_, 100 MHz, ppm): 38.92 (C-1), 27.24 (C-2), 78.79 (C-3), 38.75 (C-4), 55.73 (C-5), 18.25 (C-6), 34.27 (C-7), 39.51 (C-8), 49.53 (C-9), 37.24 (C-10), 30.33 (C-11), 75.72 (C-12), 49.42 (C-13), 51.84 (C-14), 31.42 (C-15), 25.47 (C-16), 54.38 (C-17), 15.75 (C-18), 15.78 (C-19), 74.92 (C-20), 21.75 (C-21), 35.19 (C-22), 16.59 (C-23), 37.07 (C-24), 70.66 (C-25), 32.90 (C-26), 26.69 (C-27), 28.40 (C-28), 15.38 (C-29), 17.91 (C-30). Structure of salicylic acid: 113.93 (C-1), 117.53 (C-3), 118.78 (C-5), 130.20 (C-6), 135.18 (C-4), 161.70 (C-2), 169.68 (C-7, –C=O); MS: *m*/*z* 579.35 [M − H]^+^.

*20(R)-Pananxadiol-3β-yl-2′-acetylbenzoate-12β-yl-2′-hydroxybenzoate* (**1b**, C_46_H_62_O_8_). White solid, melting point: 198–200 °C, ^1^H-NMR (CDCl_3_, 400 MHz, ppm): δ 0.65 (s, 3H), 0.91 (s, 6H), 0.93 (s, 3H), 0.97 (s, 3H), 0.99 (s, 3H), 1.05 (s, 3H), 1.15 (s, 3H), 4.69–4.73 (dd, *J*_1,3_ = 12.0 Hz, *J*_1,2_ = 4.0 Hz, 1H, H-3), 6.85–6.89 (t, *J*_1,2_ = 8.0 Hz, *J*_1,3_ = 16.0 Hz, 1H, Ar-H), 6.96–6.98 (d, *J*= 8.0 Hz, 1H, Ar-H), 7.08–7.10 (d, *J* = 8.0 Hz, 1H, Ar-H), 7.27–7.31 (t, *J*_1,3_ = 16.0 Hz, *J*_1,2_ = 8.0 Hz, 1H, Ar-H), 7.41–7.44 (t, *J*_1,3_ = 12.0 Hz, *J*_1,2_ = 6.2 Hz, 1H, Ar-H), 7.51–7.55 (t, *J*_1,3_ = 16.0 Hz, *J*_1,3_ = 8.0 Hz, 1H, Ar-H), 7.86–7.88 (d, *J* = 8.0 Hz, 1H, Ar-H), 7.96–7.98 (d, *J* = 8.0 Hz, 1H, Ar-H), 11.20 (s, 1H, Ar-OH); ^13^C-NMR (CDCl_3_, 100 MHz, ppm): 38.56 (C-1), 28.22 (C-2), 81.42 (C-3), 38.32 (C-4), 56.00 (C-5), 18.25 (C-6), 33.04 (C-7), 39.65 (C-8), 49.55 (C-9), 37.36 (C-10), 30.42 (C-11), 75.72 (C-12), 49.12 (C-13), 51.89 (C-14), 31.28 (C-15), 25.57 (C-16), 54.53 (C-17), 15.97 (C-18), 16.71 (C-19), 75.02 (C-20), 21.69 (C-21), 34.30 (C-22), 16.91 (C-23), 37.14 (C-24), 70.78 (C-25), 32.18 (C-26), 26.78 (C-27), 28.52 (C-28), 15.80 (C-29), 17.98 (C-30). Structure of salicylic acid: 114.04 (C-1), 117.66 (C-3), 118.88 (C-5), 130.29 (C-6), 135.29 (C-4), 161.84 (C-2), 169.75 (C-7, –C=O); Structure of acetylsalicylic acid: 123.88 (C-8), 124.06 (C-7), 126.01 (C-6), 131.42 (C-5), 133.63 (C-4), 150.90 (C-3), 164.05 (C-2), 169.80 (C-1, C=O), 21.27 (C-9, –CH_3_). MS: *m*/*z* 742.98 [M + H]^+^.

*20(R)-Pananxadiol-3β, 12β-di-yl-2′-acetylbenzoate* (**2b**, C_48_H_64_O_9_). White solid, melting point: 238–240 °C, ^1^H-NMR (CDCl_3_, 400 MHz, ppm): δ 0.74 (s, 3H), 0.91 (s, 3H), 0.92 (s, 3H), 0.96 (s, 3H), 0.98 (s, 3H), 1.01 (s, 3H), 1.03 (s, 3H), 1.13 (s, 3H), 4.69–4.73 (dd, *J*_1,3_ = 12.0 Hz, *J*_1,2_ = 4.0 Hz, 1H, H-3), 5.16–5.22 (td, *J*_1,2_ = 8.0 Hz, *J*_1,3_ = 12.0 Hz, 1H, H-12), 7.08–7.10 (dd, *J*_1,3_ = 8.0 Hz, 2H, Ar-H), 7.27–7.31 (td, *J*_1,3_ = 8.0 Hz, *J*_1,2_ = 4.0 Hz, 2H, Ar-H), 7.51–7.55 (td, *J*_1,3_ = 8.0 Hz, *J*_1,2_ = 4.0 Hz, 2H, Ar-H), 7.98–7.98 (dd, *J*_1,3_ = 8.0 Hz, *J*_1,2_ = 4.0 Hz, 1H, Ar-H), 8.06–8.09 (dd, *J*_1,3_ = 8.0 Hz, *J*_1,3_ = 4.0 Hz, 1H, Ar-H); ^13^C-NMR (CDCl_3_, 100 MHz, ppm): 38.52 (C-1), 28.21 (C-2), 81.46 (C-3), 38.32 (C-4), 56.00 (C-5), 18.25 (C-6), 34.35 (C-7), 39.67 (C-8), 49.63 (C-9), 37.31 (C-10), 30.68 (C-11), 75.41 (C-12), 45.30 (C-13), 52.06 (C-14), 30.79 (C-15), 25.80 (C-16), 53.63 (C-17), 16.02 (C-18), 16.63 (C-19), 75.18 (C-20), 19.29 (C-21), 34.60 (C-22), 16.90 (C-23), 37.16 (C-24), 70.70 (C-25), 33.00 (C-26), 27.22 (C-27), 28.51 (C-28), 15.89 (C-29), 18.20 (C-30); Structure of acetylsalicylic acid: 123.86, 123.96 (C-8), 124.07, 124.60 (C-7), 125.84, 125.98 (C-6), 131.01, 131.42 (C-5), 133.45, 133.59 (C-4), 150.87, 150.89 (C-3), 163.60, 164.03 (C-2), 169.64, 169.77 (C-1, C=O), 21.31, 21.25 (C-9, –CH_3_). MS: *m*/*z* 785.02 [M + H]^+^.

*20(R)-Pananxadiol-12β-hydroxy-3β-yl-2′-acetylbenzoate* (**3b**, C_39_H_58_O_6_). White solid, melting point: 243–245 °C, ^1^H-NMR (CDCl_3_, 400 MHz, ppm): δ 0.89 (s, 3H), 0.91 (s, 3H), 0.94 (s, 3H), 0.97 (s, 3H), 0.99 (s, 3H), 1.19 (s, 3H), 1.22 (s, 3H), 1.27 (s, 3H), 3.52–3.58 (td, *J*_1,3_ = 8.0 Hz, *J*_1,2_ = 4.0 Hz, 1H, H-12), 4.67–4.71 (dd, *J*_1,3_ = 12.0 Hz, *J*_1,2_ = 4.0 Hz, 1H, H-3), 7.09–7.11 (d, *J*_1,2_ = 8.0 Hz, 1H, Ar-H), 7.28–7.32 (dd, *J*_1,3_ = 12.0 Hz, *J*_1,2_ = 8.0 Hz, 1H, Ar-H), 7.52–7.56(td, *J*_1,3_ = 8.0 Hz, *J*_1,2_ = 4.0 Hz, 1H, Ar-H), 7.97–7.99 (dd, *J*_1,3_ = 8.0 Hz, *J*_1,2_ = 3.6 Hz, 1H, Ar-H); ^13^C-NMR (CDCl_3_, 100 MHz, ppm): 38.77 (C-1), 27.30 (C-2), 81.76 (C-3), 38.34 (C-4), 56.23 (C-5), 18.36 (C-6), 34.98 (C-7), 39.99 (C-8), 50.03 (C-9), 37.24 (C-10), 30.71 (C-11), 70.04 (C-12), 49.32 (C-13), 51.35 (C-14), 31.28 (C-15), 25.32 (C-16), 54.88 (C-17), 16.37 (C-18), 16.42 (C-19), 76.67 (C-20), 19.57 (C-21), 35.90 (C-22), 16.89 (C-23), 36.60 (C-24), 73.25 (C-25), 33.17 (C-26), 27.21 (C-27), 28.24 (C-28), 15.80 (C-29), 17.19 (C-30). Structure of acetylsalicylic acid: 123.89 (C-8), 124.19 (C-7), 126.05 (C-6), 131.49 (C-5), 133.61 (C-4), 150.88 (C-3), 164.22 (C-2), 169.80 (C-1, C=O), 21.29 (C-9, CH_3_). MS: *m*/*z* 622.87 [M + H]^+^.

*20(R)-Pananxadiol-3β-o-acetyl-12β-yl-2′-hydroxybenzoate* (**4b**, C_39_H_58_O_6_). White solid, melting point: 238–240 °C, ^1^H-NMR (CDCl_3_, 400 MHz, ppm): δ 0.85 (s, 3H), 0.86 (s, 3H), 0.87 (s, 3H), 0.90 (s, 3H), 0.96 (s, 3H), 0.98 (s, 3H), 1.03 (s, 3H), 1.15 (s, 3H), 4.47–4.51 (dd, *J*_1,3_ = 12.0 Hz, *J*_1,2_ = 4.0 Hz, 1H, H-3), 6.84–6.88 (t, *J*_1,3_ = 16.0 Hz, *J*_1,2_ = 8.0 Hz, 1H, Ar-H), 6.95–6.97 (d, *J*_1,2_ = 8.0 Hz, 1H, Ar-H), 7.40–7.44 (td, *J*_1,2_ = 4.0 Hz, *J*_1,3_ = 8.0 Hz, 1H, Ar-H), 7.85–7.87 (dd, *J*_1,3_ = 8.0 Hz, *J*_1,2_ = 4.0 Hz, 1H, Ar-H), 11.19 (s, 1H, Ar-OH); ^13^C-NMR (CDCl_3_, 100 MHz, ppm): 38.55 (C-1), 28.14 (C-2), 80.74 (C-3), 38.01 (C-4), 55.91 (C-5), 18.27 (C-6), 34.31 (C-7), 39.65 (C-8), 49.54 (C-9), 37.37 (C-10), 30.43 (C-11), 75.76 (C-12), 45.08 (C-13), 51.90 (C-14), 30.71 (C-15), 23.70 (C-16), 54.54 (C-17), 15.96 (C-18), 16.66 (C-19), 75.04 (C-20), 19.33 (C-21), 35.38 (C-22), 16.72 (C-23), 37.12 (C-24), 70.79 (C-25), 33.05 (C-26), 26.80 (C-27), 28.52 (C-28), 15.89 (C-29), 17.99 (C-30), 21.43 (–CH_3_), 171.02 (–C=O); Structure of salicylic acid: 114.06 (C-1), 117.67 (C-3), 118.88 (C-5), 130.30 (C-6), 135.29 (C-4), 161.85 (C-2), 169.76 (C-7, –C=O).

*20(R)-Pananxadiol-3β-hydroxy-12β-yl-2′-acetylbenzoate* (**5b**, C_39_H_58_O_6_). White solid, melting point: 226–228 °C, ^1^H-NMR (CDCl_3_, 400 MHz, ppm): δ 0.74 (s, 3H), 0.77 (s, 3H), 0.85 (s, 3H), 0.98 (s, 6H), 1.00 (s, 3H), 1.01 (s, 3H), 1.11 (s, 3H), 3.17–3.21 (dd, *J*_1,3_ = 12.0 Hz, *J*_1,2_ = 4.0 Hz, 1H, H-3), 5.13–5.20 (td, *J*_1,3_ = 12.0 Hz, *J*_1,2_ = 8.0 Hz, 1H, H-12), 7.07–7.09 (d, *J* = 8.0 Hz, 1H, Ar-H), 7.28–7.31 (t, *J*_1,2_ = 6.0 Hz, *J*_1,3_ = 12.0 Hz, 1H, Ar-H), 7.50–7.54 (td, *J*_1,3_ = 8.0 Hz, *J*_1,2_ = 4.0 Hz, 1H, Ar-H), 8.05–8.08 (dd, *J*_1,2_ = 4.0 Hz, *J*_1,3_ = 8.0 Hz, 1H, Ar-OH); ^13^C-NMR (CDCl_3_, 100 MHz, ppm): 39.05 (C-1), 28.12 (C-2), 78.95 (C-3), 38.85 (C-4), 55.90 (C-5), 18.39 (C-6), 33.04 (C-7), 39.67 (C-8), 49.78 (C-9), 37.33 (C-10), 30.44 (C-11), 75.53 (C-12), 45.34 (C-13), 52.17 (C-14), 30.88 (C-15), 25.86 (C-16), 53.63 (C-17), 15.90 (C-18), 15.99 (C-19), 75.24 (C-20), 22.94 (C-21), 34.47 (C-22), 16.65 (C-23), 37.23 (C-24), 70.72 (C-25), 32.09 (C-26), 27.40 (C-27), 28.55 (C-28), 15.51 (C-29), 18.29 (C-30); Structure of acetylsalicylic acid: 123.98 (C-8), 124.65 (C-7), 125.89 (C-6), 132.16 (C-5), 133.48 (C-4), 150.87 (C-3), 163.66 (C-2), 169.67 (C-1, C=O), 21.33 (C-9, CH_3_).

*20(R)-Dammarane-20,25-diol-12β-o-acetyl-3β-yl-2′-hydroxybenzoate* (**6b**, C_39_H_60_O_7_). White solid, melting point: 278–280 °C, ^1^H-NMR (CDCl_3_, 400 MHz, ppm): δ 0.94 (s, 6H), 0.98 (s, 3H), 1.02 (s, 3H), 1.04 (s, 3H), 1.11 (s, 3H),1.22 (s, 6H), 4.74–4.78 (dd, *J*_1,3_ = 8.0 Hz, *J*_1,2_ = 4.0 Hz, 2H, H-12, H-3), 6.85–6.89 (t, *J*_1,3_ = 16.0 Hz, *J*_1,2_ = 8.0 Hz, 1H, Ar-H), 6.97–6.99 (d, *J*_1,2_ = 8.0 Hz, 1H, Ar-H), 7.42–7.47 (td, *J*_1,3_ = 12.0 Hz, *J*_1,2_ = 4.0 Hz, 1H, Ar-H), 7.80–7.83 (dd, *J*_1,3_ = 8.0 Hz, *J*_1,2_ = 4.0 Hz, 1H, Ar-H), 10.92 (s, 1H, Ar-OH); ^13^C-NMR (CDCl_3_, 100 MHz, ppm): 39.92 (C-1), 28.29 (C-2), 82.08 (C-3), 38.64 (C-4), 56.10 (C-5), 18.10 (C-6), 34.64 (C-7), 42.77 (C-8), 49.16 (C-9), 38.41 (C-10), 31.83 (C-11), 76.64 (C-12), 49.05 (C-13), 52.94 (C-14), 31.72 (C-15), 27.36 (C-16), 50.16 (C-17), 16.42 (C-18), 16.84 (C-19), 73.87 (C-20), 23.16 (C-21), 44.59 (C-22), 18.28 (C-23), 45.66 (C-24), 71.27 (C-25), 29.85 (C-26), 29.60 (C-27), 28.52 (C-28), 15.64 (C-29), 17.78 (C-30), 21.71 (–CH_3_), 169.94 (–C=O). Structure of salicylic acid: 113.17 (C-1), 117.75 (C-3), 119.18 (C-5), 129.85 (C-6), 135.62 (C-4), 161.89 (C-2), 169.75 (–C=O); MS: *m*/*z* 640.89 [M + H]^+^.

*20(R)-Dammarane-20,25-diol-12β-o-acetyl-3β-yl-2′-acetylbenzoate* (**7b**, C_39_H_62_O_8_). White solid, melting point: 268–270 °C, ^1^H-NMR (CDCl_3_, 400 MHz, ppm): δ 0.91 (s, 6H), 0.96 (s, 6H), 1.02 (s, 3H), 1.10 (s, 3H), 1.21 (s, 6H), 4.68–4.78 (m, 2H, H-12, H-3), 7.08–7.70 (d, *J*_1,2_ = 8.0 Hz, 1H, Ar-H), 7.27–7.31 (t, *J*_1,3_ = 8.0 Hz, *J*_1,2_ = 4.0 Hz, 1H, Ar-H), 7.51–7.55 (td, *J*_1,3_ = 8.0 Hz, *J*_1,2_ = 4.0 Hz,1H, Ar-H), 7.95–7.98 (dd, *J*_1,3_ =8.0 Hz, *J*_1,2_ = 4.0 Hz, 1H, Ar-H); ^13^C-NMR (CDCl_3_, 100 MHz, ppm): 38.32 (C-1), 28.19 (C-2), 81.35 (C-3), 38.66 (C-4), 56.12 (C-5), 18.07 (C-6), 34.63 (C-7), 39.87 (C-8), 50.15 (C-9), 37.23 (C-10), 32.05 (C-11), 76.62 (C-12), 49.11 (C-13), 52.89 (C-14), 31.79 (C-15), 27.33 (C-16), 51.35 (C-17), 16.42 (C-18), 16.87 (C-19), 73.85 (C-20), 22.81 (C-21), 44.55 (C-22), 18.24 (C-23), 45.62 (C-24), 71.21 (C-25), 29.56 (C-26), 29.48 (C-27), 29.31 (C-28), 15.60 (C-29), 17.74 (C-30), 21.69 (–CH_3_), 169.82 (–C=O). Structure of acetylsalicylic acid: 169.72 (C-1, –C=O), 164.02 (C-2), 150.90 (C-3), 133.65 (C-4), 131.41 (C-5), 126.01 (C-6), 124.04 (C-7), 123.88 (C-8), 21.25 (C-9, –CH_3_). MS: *m*/*z* 682.93 [M + H]^+^.

*20(R)-Dammarane-12β,20,25-diol-3β-yl-2′-acetylbenzoate* (**8b**, C_37_H_60_O_7_). White solid, melting point: 254–256 °C, ^1^H-NMR (CDCl_3_, 400 MHz, ppm): δ 0.90 (s, 3H), 0.91 (s, 3H), 0.94 (s, 3H), 0.97 (s, 3H), 1.01 (s, 3H), 1.15 (s, 3H), 1.24 (s, 6H), 3.62–3.68 (td, *J*_1,3_ = 12.0 Hz, *J*_1,2_ = 4.0 Hz, 1H, H-12), 4.68–4.72 (dd, *J*_1,3_ = 12.0 Hz,, *J*_1,2_ = 8.0 Hz, 1H, H-3), 7.09–7.11 (d, *J* = 8.0 Hz, 1H, Ar-H), 7.28–7.32 (t, *J*_1,3_ = 8.0 Hz, *J*_1,2_ = 3.5 Hz, 1H, Ar-H), 7.52–7.56 (td, *J*_1,3_ = 8.0 Hz, *J*_1,2_ = 4.0 Hz, 1H, Ar-H), 7.97–7.99 (dd, *J*_1,3_ = 8.0 Hz, *J*_1,2_ = 4.0 Hz, 1H); ^13^C-NMR (CDCl_3_, 100 MHz, ppm): 39.81 (C-1), 28.23 (C-2), 81.59 (C-3), 38.33 (C-4), 56.17 (C-5), 17.85 (C-6), 34.83 (C-7), 39.91 (C-8), 50.07 (C-9), 37.20 (C-10), 31.36 (C-11), 71.34 (C-12), 48.47 (C-13), 51.78 (C-14), 51.78 (C-15), 31.16 (C-16), 50.13 (C-17), 16.39 (C-18), 16.91 (C-19), 74.80 (C-20), 21.99 (C-21), 42.98 (C-22), 18.30 (C-23), 44.23 (C-24), 71.15 (C-25), 29.56 (C-26), 29.41 (C-27), 28.46 (C-28), 15.81 (C-29), 17.25 (C-30). Structure of acetylsalicylic acid: 169.82 (C-1, –C=O), 164.16 (C-2), 150.89 (C-3), 133.68 (C-4), 131.50 (C-5), 126.07 (C-6), 124.09 (C-7), 123.90 (C-8), 21.30 (C-9, –CH_3_); MS: *m*/*z* 640.89 [M + H]^+^.

### 4.4. Cytotoxicity Assay

In the study, five cancer cell lines (human colon cancer cells (HT-29), gastric cancer cells (BGC-823), cervical cancer cells (Hela), human breast cancer cells (MCF-7), and human lung cancer cells A549)), and two normal cell lines (human gastric epithelial cells (GES-1) and human ovarian epithelial cells (IOSE144)) were used for the evaluation of biological activity. IOSE144 cells were donated by Professor Piao Huri of Yanbian University, all other cells were purchased from the American Type Culture Collection (ATCC, Manassas, VA, USA). Cells were cultured in Dulbecco’s Modified Eagle’s Medium (DMEM), supplemented with 10% heat-inactivated fetal bovine serum (FBS), 1% 100 μg/mL penicillin and 100 μg/mL streptomycin in a humidified atmosphere containing 5% CO_2_ at 37 °C. Cell viability was measured using the MTT assay. Cells were seeded in 96 well microtiter plates in a volume of 100 μL medium. All compounds tested were dissolved and further diluted in DMSO. After overnight incubation, cells were treated with different test concentrations or carrier solvents alone in a final volume of 100 μL with five replicates each. The concentration of DMSO did not exceed 0.1%. Cells were treated with compound (80, 40, 20, 10, 5 μM) for 48 h. Then, 10 μL of MTT (5 mg/mL) was added to each well and the cells were incubated at 37 °C in the dark for 4 h. Supernatants were removed and formazan crystals were dissolved in DMSO (100 μL/well). The solution was agitated for 10 min, and the absorbance was measured at 490 nm using microplate reader (imark, BIO-RAD, Hercules, CA, USA) to calculate the 50% inhibitory concentration (IC_50_). Ginsenoside-Rg_3_, panaxadiol sapogenin (PD), AD-2 (25-OH-PPD) and AD-1 (25-OCH_3_-PPD) were used as standard reference compounds.

### 4.5. Cell Cycle Distribution Assay

MCF-7 cells treated with 30 μM of compound **3b** were incubated for 24 h in CO_2_ incubator. AD-1 was tested as a positive control, and the experimental concentration of AD-1 was 30 μM. Cells were trypsinized, harvested, washed with PBS, and fixed with 75% ethanol overnight at −20 °C. Next, fixed cells were washed with PBS and stained for 4 h in the dark with a PI solution consisting of 50 μg/mL PI, 50 μg/mL RNase, 0.1% sodium citrate and 0.1% Triton X-100 (pH 8.0) [22]. Cell cycle distribution was determined using FACScan (Becton Dickinson, Franklin Lakes, NJ, USA).

### 4.6. Wound Healing Assay

MCF-7 cells were seeded in 10-cm dishes at a concentration of 1 × 10^5^ cells/ mL and grown overnight. Then, a wound was created in the monolayer of cells by scratching the monolayer with a sharp tip followed by another 24 h of incubation in the presence of 30 μM of compound **3b** or 0.1% DMSO under serum-free conditions, 30 μM of AD-1 as a positive control. The gap created was measured under a microscope to provide an indication of the wound healing capability of the cells [23].

### 4.7. Statistical Analysis

Data are presented as the mean ± S.E.M. Statistical analysis was performed using IBM SPSS 19.0 (IBM, Chicago, IL, USA) software and GraphPad Prism 5 (GraphPad software, San Diego, CA, USA) software.

## Figures and Tables

**Table 1:** Products of ginsenosides with salicylic acid and acetylsalicylic acid.

Compound	Structure	Compound	Structure
**1a**	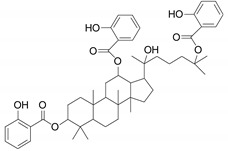	**11a**	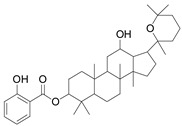
**2a**	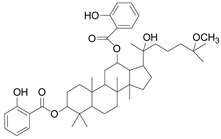	**12a**	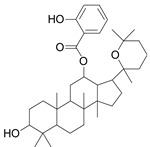
**3a**	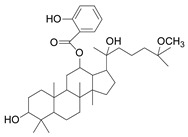	**1b**	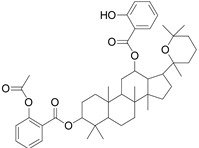
**4a**	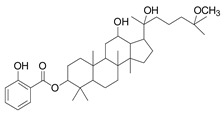	**2b**	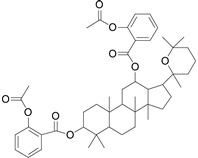
**5a**	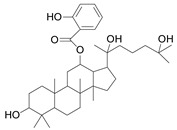	**3b**	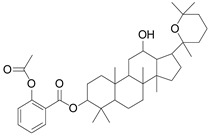
**6a**	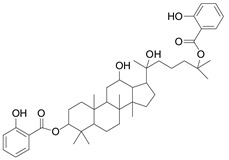	**4b**	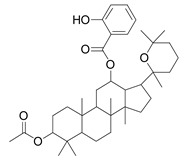
**7a**	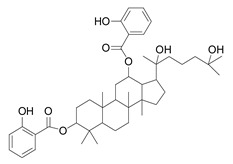	**5b**	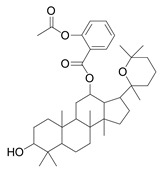
**8a**	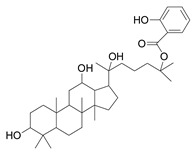	**6b**	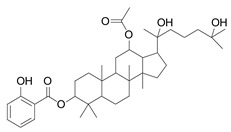
**9a**	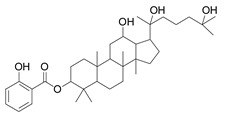	**7b**	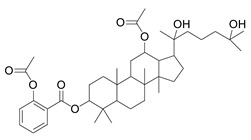
**10a**	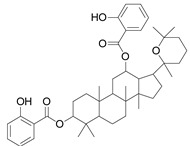	**8b**	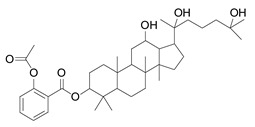

**Table 2:** Antitumor activity and cytotoxicity of ginsenoside derivatives (IC_50_, μM).

Compound	Cell Lines						
	HT-29	BGC-823	Hela	MCF-7	A549	GES-1	IOSE144
**1a**	9.23 ± 0.08	11.84 ± 0.21	3.23 ± 0.34	20.31 ± 0.11	15.69 ± 0.10	>100	>100
**2a**	32.58 ± 0.23	40.85 ± 0.45	16.53 ± 0.23	25.69 ± 0.43	26.45 ± 0.14	>100	>100
**3a**	10.53 ± 0.25	15.12 ± 0.24	9.78 ± 0.03	5.34 ± 0.04	12.95 ± 0.25	>100	>100
**4a**	50.64 ± 1.03	13.25 ± 0.18	19.26 ± 0.14	11.13 ± 0.35	35.62 ± 0.56	>100	>100
**5a**	18.35 ± 0.47	60.52 ± 1.11	21.56 ± 1.08	18.46 ± 0.23	22.51 ± 0.46	>100	>100
**6a**	>100	>100	20.36 ± 0.36	>100	19.35 ± 0.59	>100	>100
**7a**	22.69 ± 0.46	70.56 ± 2.12	35.23 ± 1.24	29.10 ± 0.69	8.52 ± 0.15	>100	>100
**8a**	>100	>100	>100	50.69 ± 1.34	47.15 ± 0.59	>100	>100
**9a**	11.54 ± 0.23	26.52 ± 0.26	37.26 ± 0.39	50.44 ± 0.11	35.86 ± 0.41	>100	>100
**10a**	30.25 ± 0.02	>100	>100	41.38 ± 0.48	29.46 ± 0.61	>100	>100
**11a**	26.52 ± 0.56	41.86 ± 0.43	>100	11.54 ± 1.47	23.78 ± 1.36	>100	>100
**12a**	>100	>100	>100	60.14 ± 2.45	50.23 ± 0.94	>100	>100
**1b**	12.25 ± 0.28	9.65 ± 0.61	11.34 ± 0.14	7.65 ± 0.29	13.26 ± 0.11	>100	>100
**2b**	5.27 ± 0.14	8.12 ± 0.25	16.22 ± 0.34	10.25 ± 0.05	15.47 ± 0.13	>100	>100
**3b**	10.69 ± 0.12	11.24 ± 0.16	20.94 ± 0.54	2.56 ± 0.09	14.37 ± 0.04	>100	>100
**4b**	36.12 ± 0.28	50.26 ± 0.39	48.75 ± 0.37	39.41 ± 0.68	25.45 ± 0.77	>100	>100
**5b**	20.12 ± 0.74	26.24 ± 0.02	18.65 ± 0.13	24.36 ± 0.15	14.13 ± 0.52	>100	>100
**6b**	15.23 ± 0.41	40.62 ± 1.05	48.31 ± 2.16	>100	>100	>100	>100
**7b**	28.56 ± 0.65	18.42 ± 0.35	19.53 ± 0.19	22.59 ± 1.02	28.44 ± 0.95	>100	>100
**8b**	13.25 ± 0.05	10.25 ± 0.29	10.03 ± 0.53	14.67 ± 0.24	6.12 ± 0.10	>100	>100
Salicylic acid *	83.16 ± 1.65	>100	>100	>100	>100	>100	>100
Acetyl-salicylic acid *	70.26 ± 0.38	87.67 ± 0.78	>100	57.12 ± 1.70	90.46 ± 2.13	>100	>100
PD *	73.44 ± 1.84	80.13 ± 0.36	72.15 ± 0.52	61.96 ± 1.63	43.13 ± 1.05	>100	>100
AD-2 *	18.69 ± 0.28	44.25 ± 1.06	48.63 ± 1.24	49.64 ± 1.35	34.26 ± 0.87	>100	>100
AD-1 *	16.58 ± 0.25	16.57 ± 0.45	12.99 ± 0.06	7.34 ± 0.19	34.32 ± 0.31	>100	>100
Rg_3_ *	>100	>100	85.23 ± 1.59	92.12 ± 2.36	>100	>100	>100

*: Positive control: known anti-cancer active compounds.

## Supplementary Materials

^1^H and ^13^C-NMR spectra of compounds.
