# Supplementary material for: Synthesis and Anticancer Activity Evaluation of Hydrolyzed Derivatives of Panaxnotoginseng Saponins
Source: Molecules. 2018 Nov 19;23(11):3021. doi: 10.3390/molecules23113021 (PMC6278399; doi:10.3390/molecules23113021)
Supplement: Supplementary file 1 [file molecules-23-03021-s001.pdf]

Supplementary Material

Synthesis and Anticancer Activity Evaluation of Hydrolyzed Derivatives of *Panaxnotoginseng* Saponins

Lei Xu [1,2], Shengnan Xiao [1], Weihui Yuan [1], Jiongmo Cui [2], Guangyue Su [1,*] and Yuqing Zhao [1,3,*]

1 School of Functional Food and Wine, Shenyang Pharmaceutical University, Shenyang 110016, China; 18246691730@163.com (L.X.); 15931605679@163.com (S.X.); 15142590591@163.com (W.Y.)

2 College of Pharmacy, Yanbian University, Yanji 133000, China; jmcui@ybu.edu.cn

3 Key Laboratory of Structure-based Drug Design and Discovery of Ministry of Education, Shenyang Pharmaceutical University, Shenyang 110016, China

* Correspondence: suggyy@163.com (G.S.); zyq4885@126.com (Y.Z.); Tel: +86-24-43520309 (G.S.); +86-24-43520303 (Y.Z.); Fax: +86-24-43520300 (Y.Z. & G.S.)

Contents

Figure S1. ^{1}H-NMR of compound **1a** 3

Figure S2. ^{13}C-NMR of compound **1a** 3

Figure S3. ^{1}H-NMR of compound **2a** 4

Figure S4. ^{13}C-NMR of compound **2a** 4

Figure S5. ^{1}H-NMR of compound **3a** 5

Figure S6. ^{13}C-NMR of compound **3a** 5

Figure S7. ^{1}H-NMR of compound **4a** 6

Figure S8. ^{13}C-NMR of compound **4a** 6

Figure S9. ^{1}H-NMR of compound **5a** 7

Figure S10. ^{13}C-NMR of compound **5a** 7

Figure S11. ^{1}H-NMR of compound **6a** 8

Figure S12. ^{13}C-NMR of compound **6a** 8

Figure S13. ^{1}H-NMR of compound **7a** 9

Figure S14. ^{13}C-NMR of compound **7a** 9

Figure S15. ^{1}H-NMR of compound **8a** .... **Error! Bookmark not defined.**

Figure S16. ^{13}C-NMR of compound **8a** .... **Error! Bookmark not defined.**

Figure S17. ^{1}H-NMR of compound **9a** 11

Figure S18. ^{13}C-NMR of compound **9a** .... **Error! Bookmark not defined.**

Figure S19. ^{1}H-NMR of compound **10a** .... **Error! Bookmark not defined.**

Figure S20. ^{13}C-NMR of compound **10a**..**Error! Bookmark not defined.**

Figure S21. ^{1}H-NMR of compound **11a** 13

Figure S22. ^{13}C-NMR of compound **11a**.. 13

Figure S23. ^{1}H-NMR of compound **12a** 14

Figure S24. ^{13}C-NMR of compound **12a**.. 14

Figure S25. ^{1}H-NMR of compound **1b** 15

Figure S26. ^{13}C-NMR of compound **1b** 15

Figure S27. ^{1}H-NMR of compound **2b** 16

Figure S28. ^{13}C-NMR of compound **2b** 16

Figure S29. ^{1}H-NMR of compound **3b** 17

Figure S30. ^{13}C-NMR of compound **3b** 17

Figure S31. ^{1}H-NMR of compound **4b** 18

Figure S32. ^{13}C-NMR of compound **4b** 18

Figure S33. ^{1}H-NMR of compound **5b** 19

Figure S34. ^{13}C-NMR of compound **5b** 19

Figure S35. ^{1}H-NMR of compound **6b** 20

Figure S36. ^{13}C-NMR of compound **6b** 20

Figure S37. ^{1}H-NMR of compound **7b** 21

Figure S38. ^{13}C-NMR of compound **7b** 21

Figure S39. ^{1}H-NMR of compound **8b** 22

Figure S40. ^{13}C-NMR of compound **8b** 22

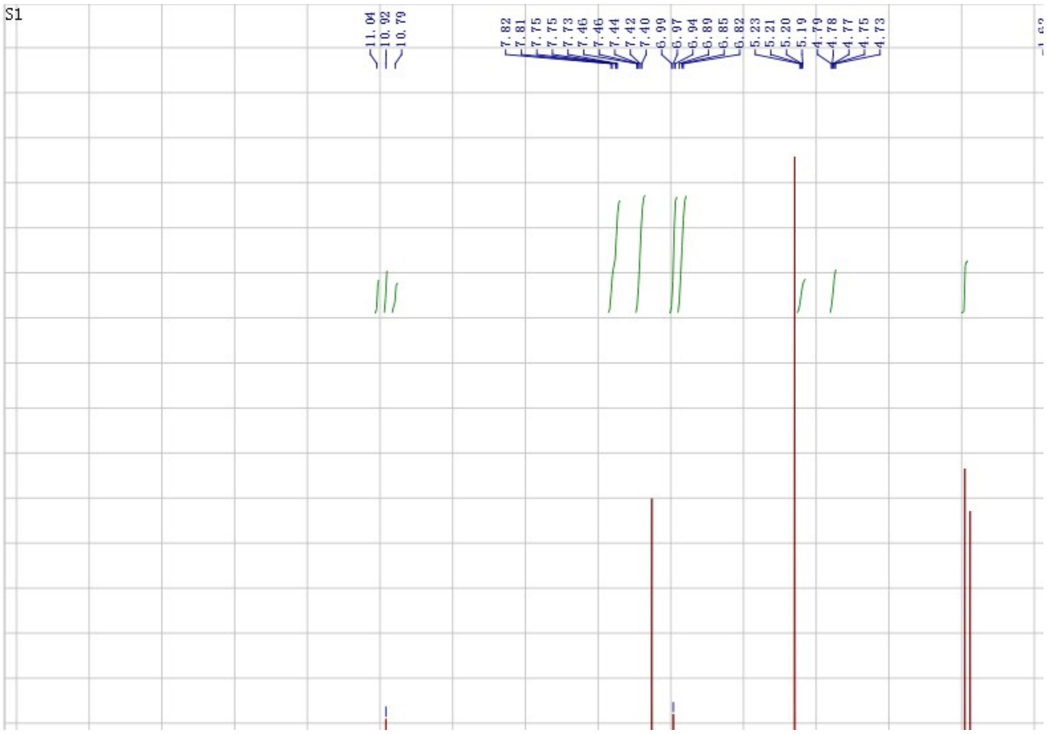

Figure S1. ^{1}H-NMR of compound **1a**

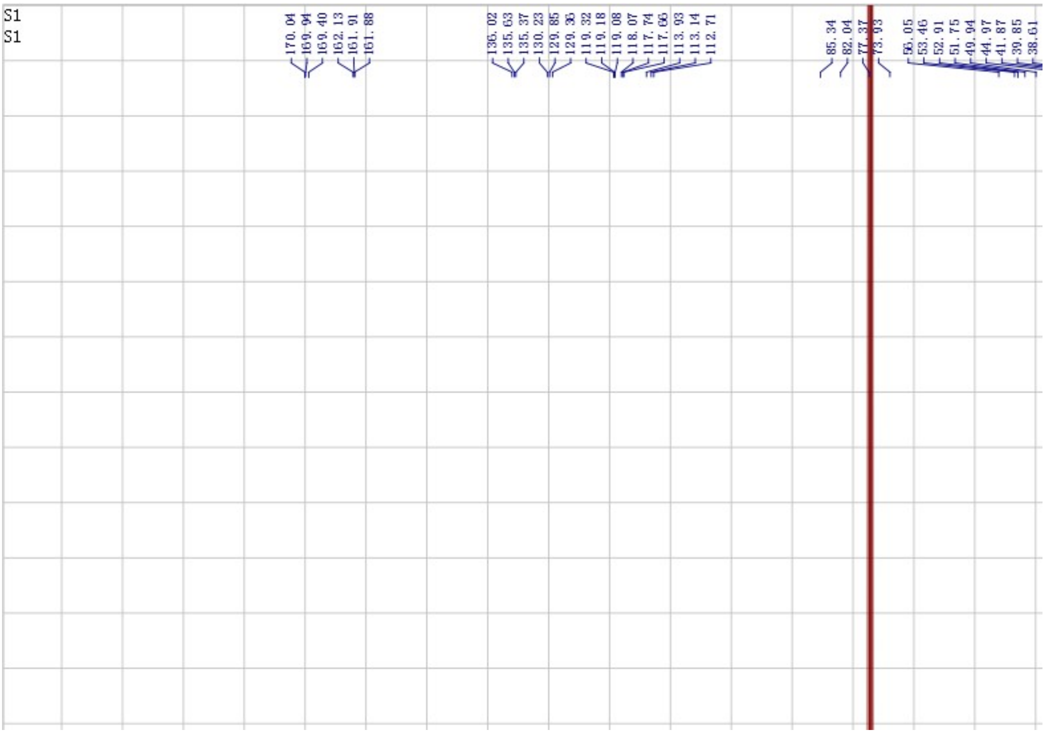

Figure S2. ^{13}C-NMR of compound **1a**

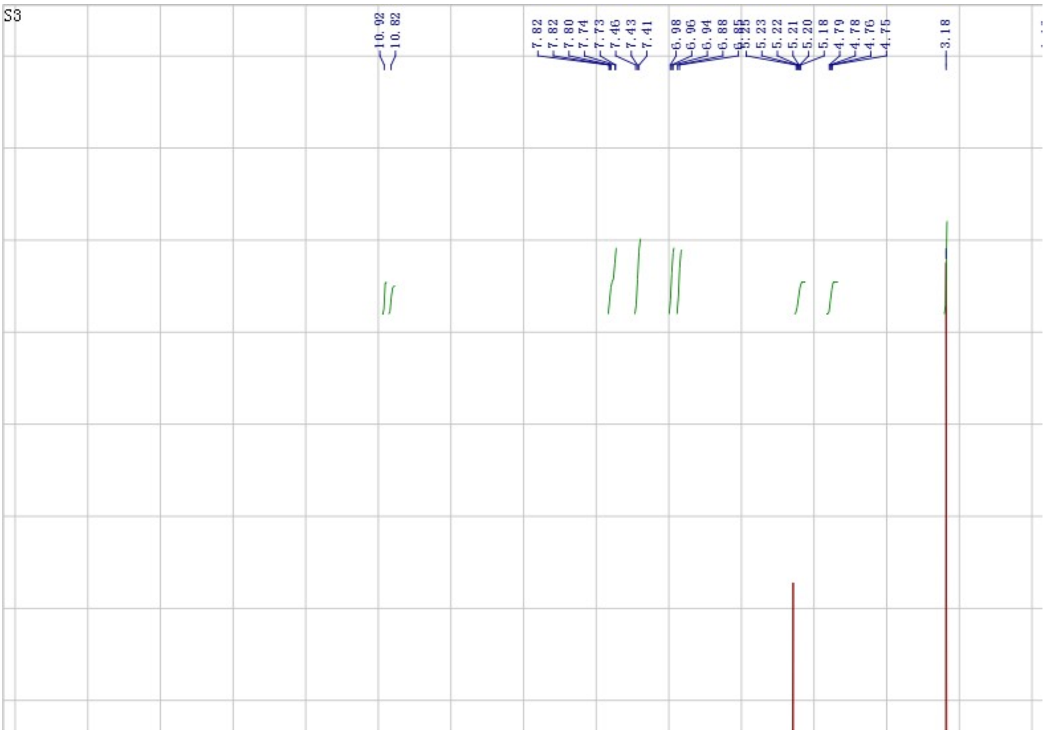

Figure S3. ^{1}H-NMR of compound **2a**

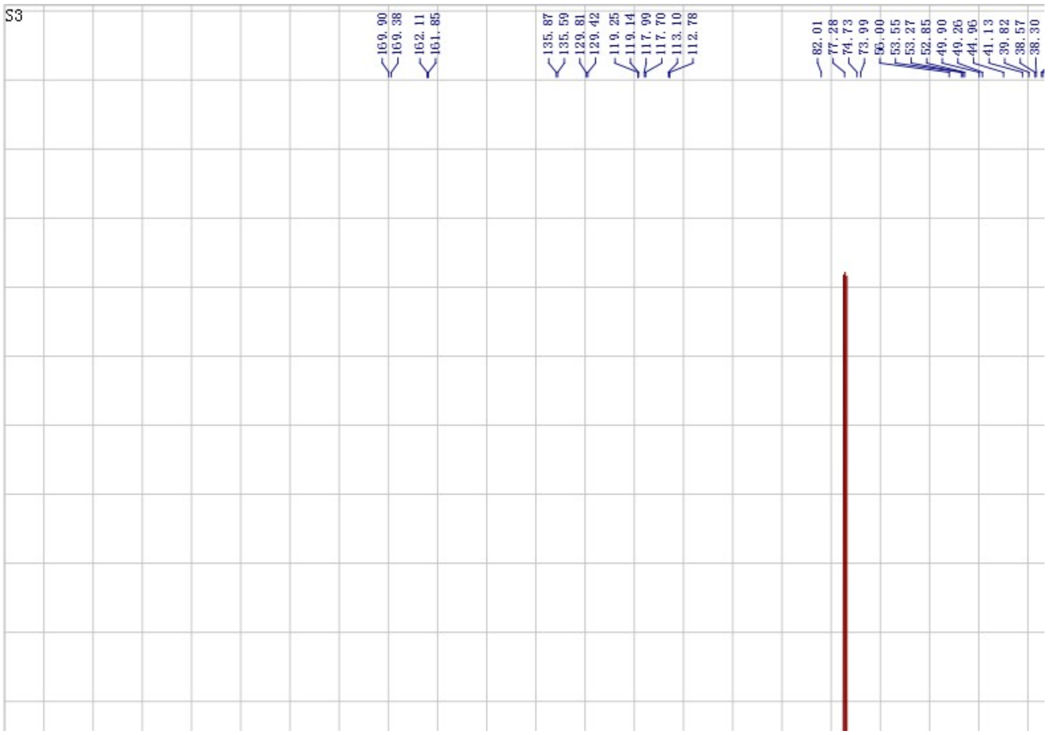

Figure S4. ^{13}C-NMR of compound **2a**

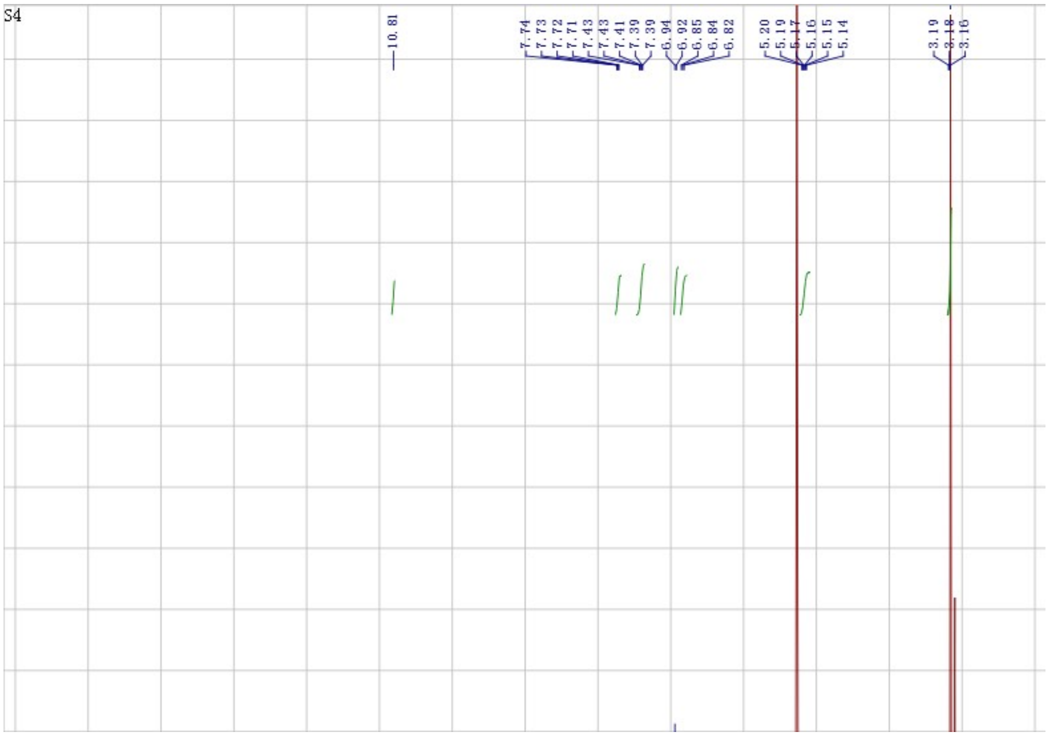

Figure S5. ^{1}H-NMR of compound **3a**

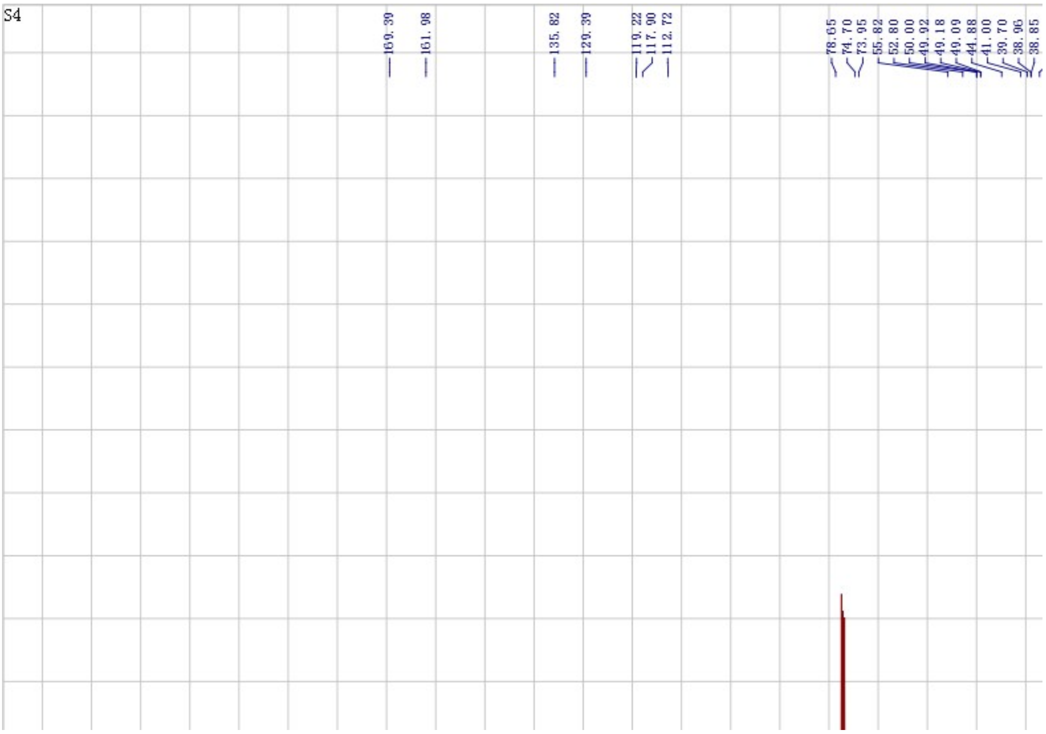

Figure S6. ^{13}C-NMR of compound **3a**

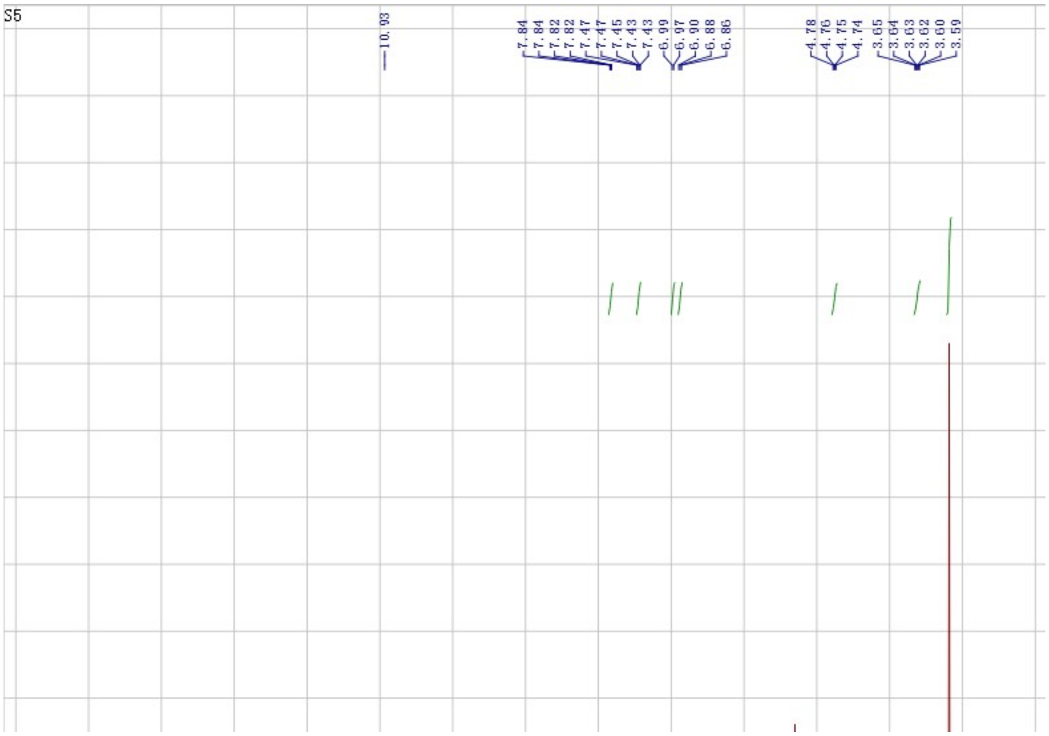

Figure S7. ¹H-NMR of compound **4a**

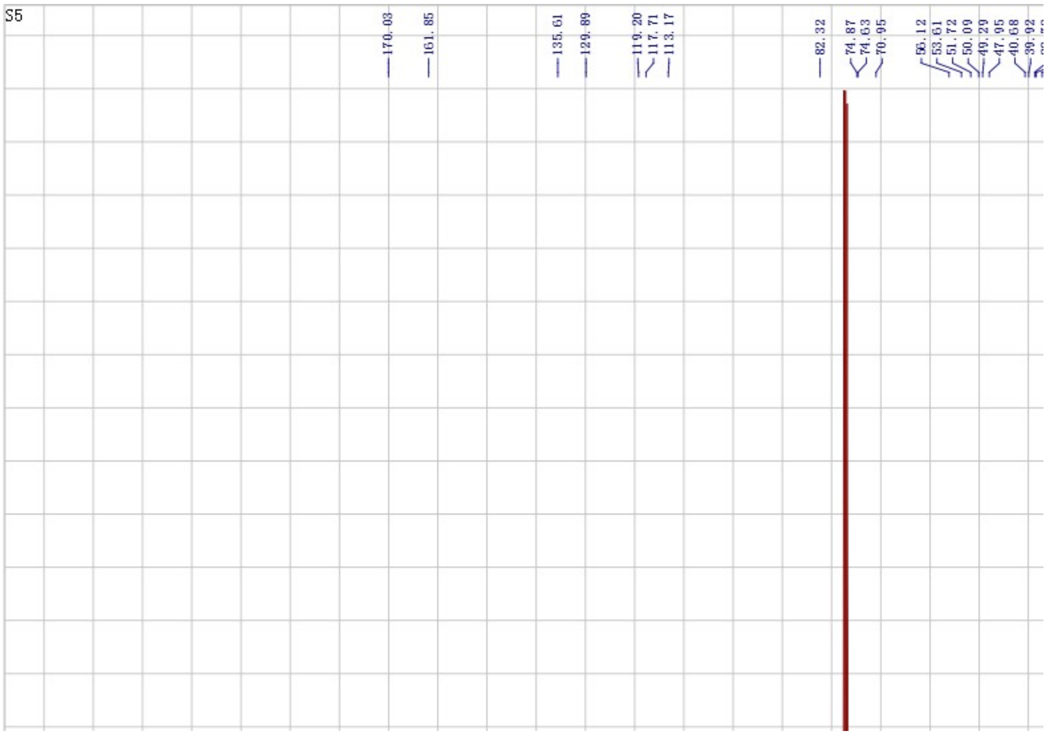

Figure S8. ¹³C-NMR of compound **4a**

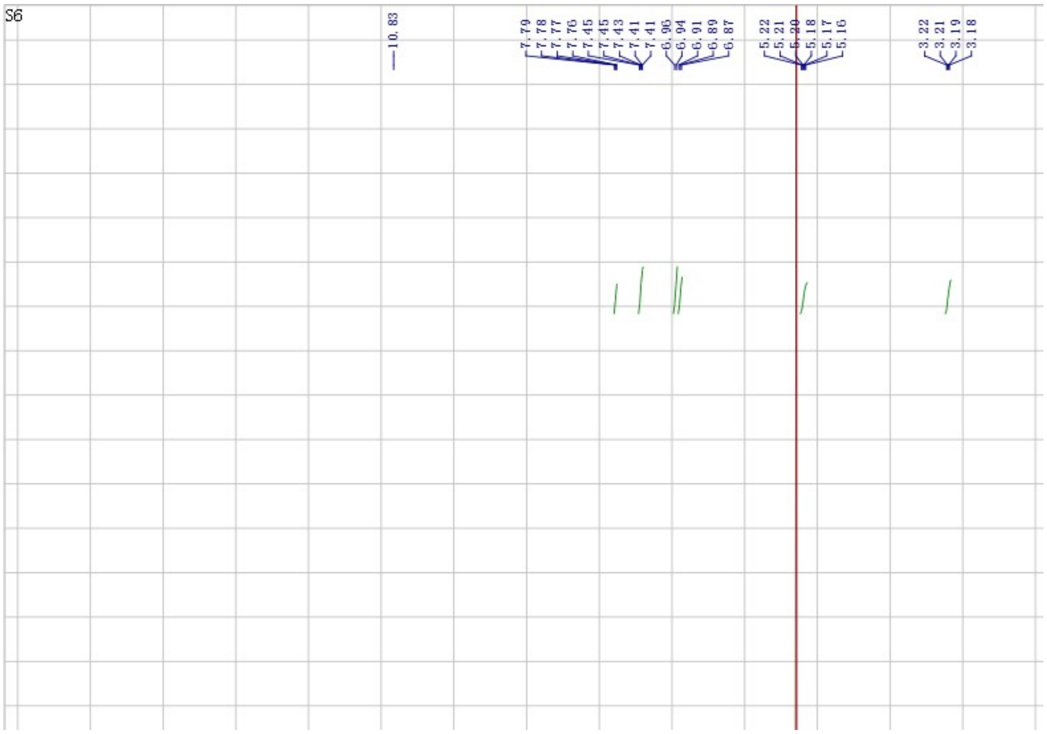

Figure S9. ^{1}H-NMR of compound **5a**

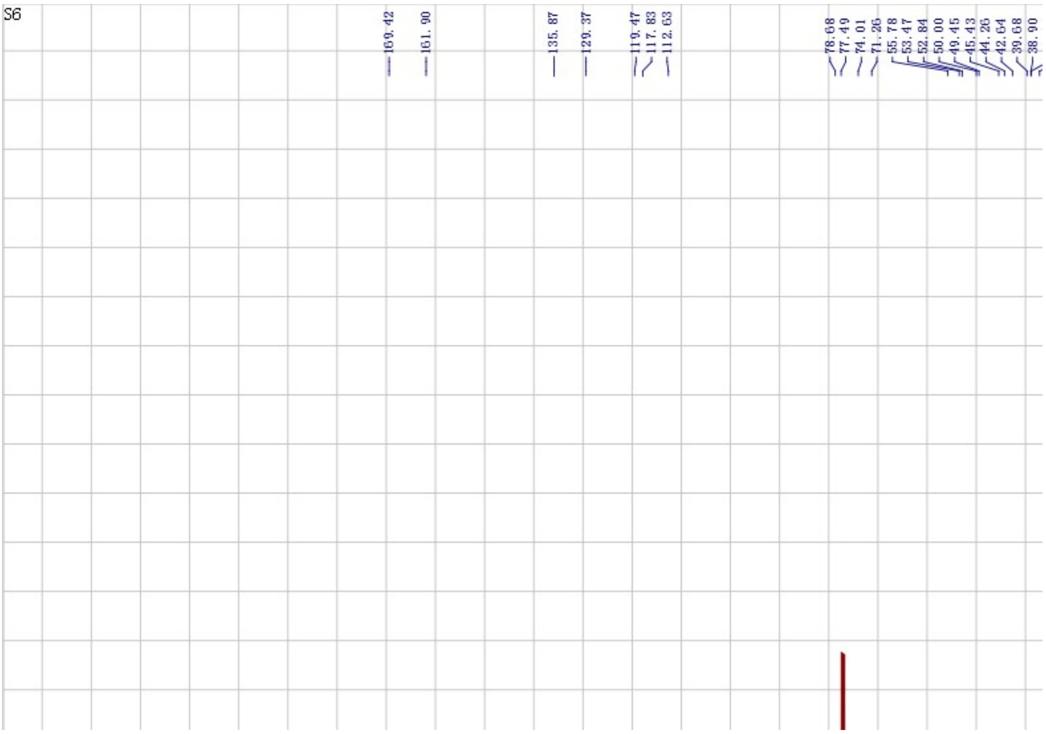

Figure S10. ^{13}C-NMR of compound **5a**

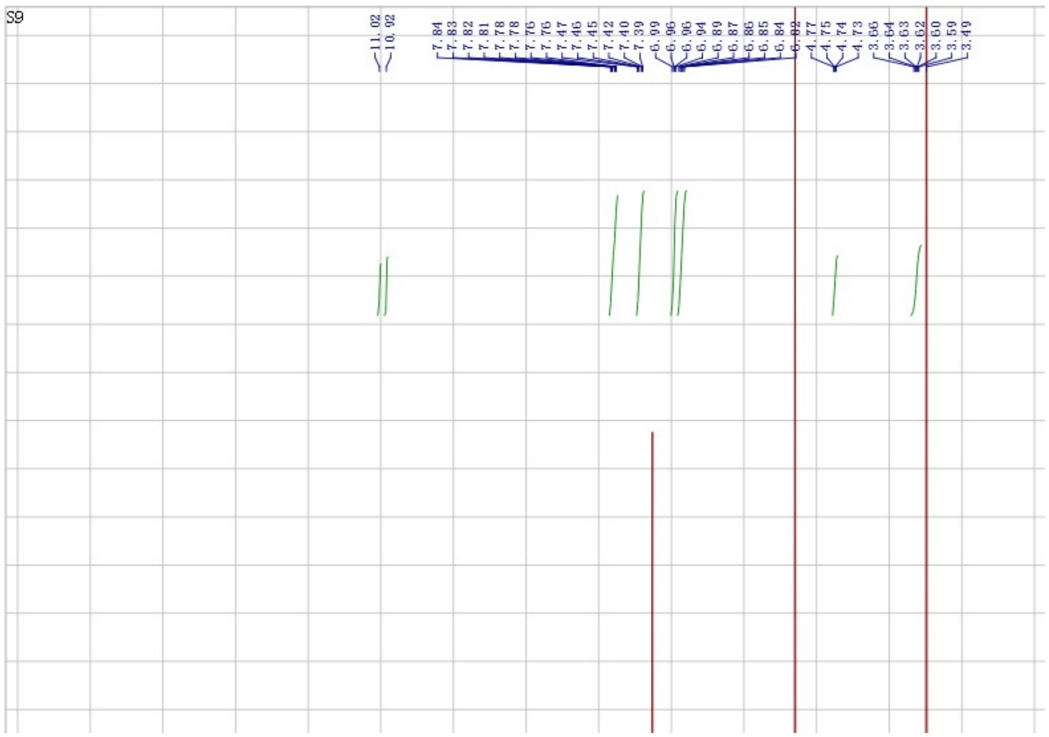

Figure S11. ¹H-NMR of compound **6a**

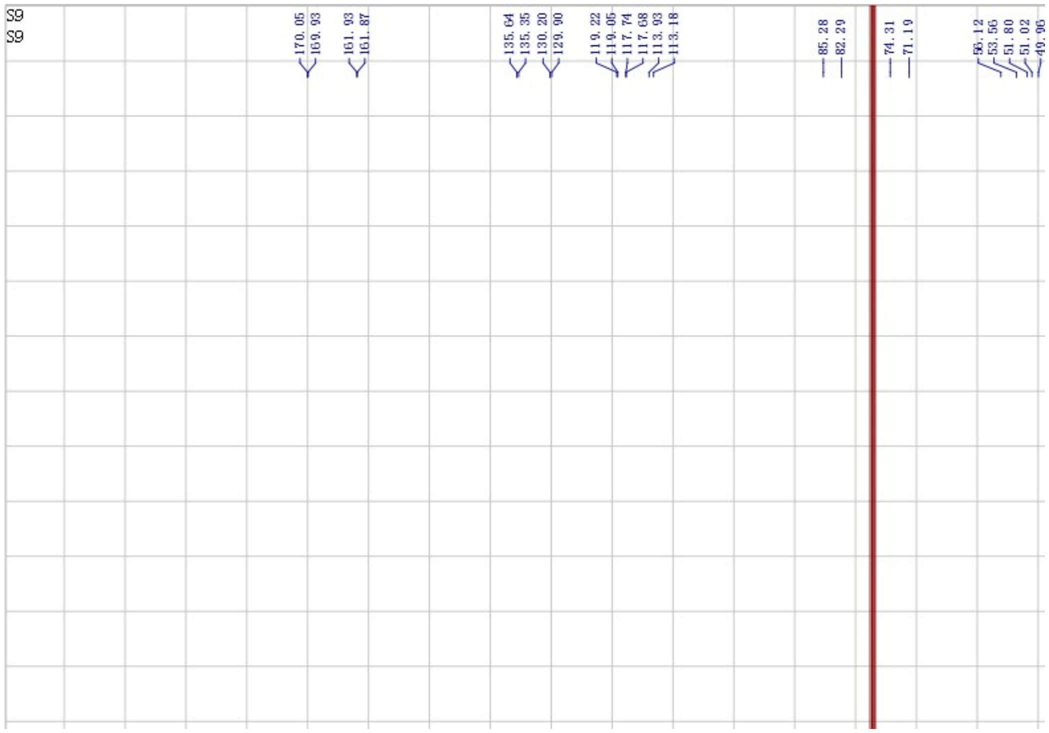

Figure S12. ¹³C-NMR of compound **6a**

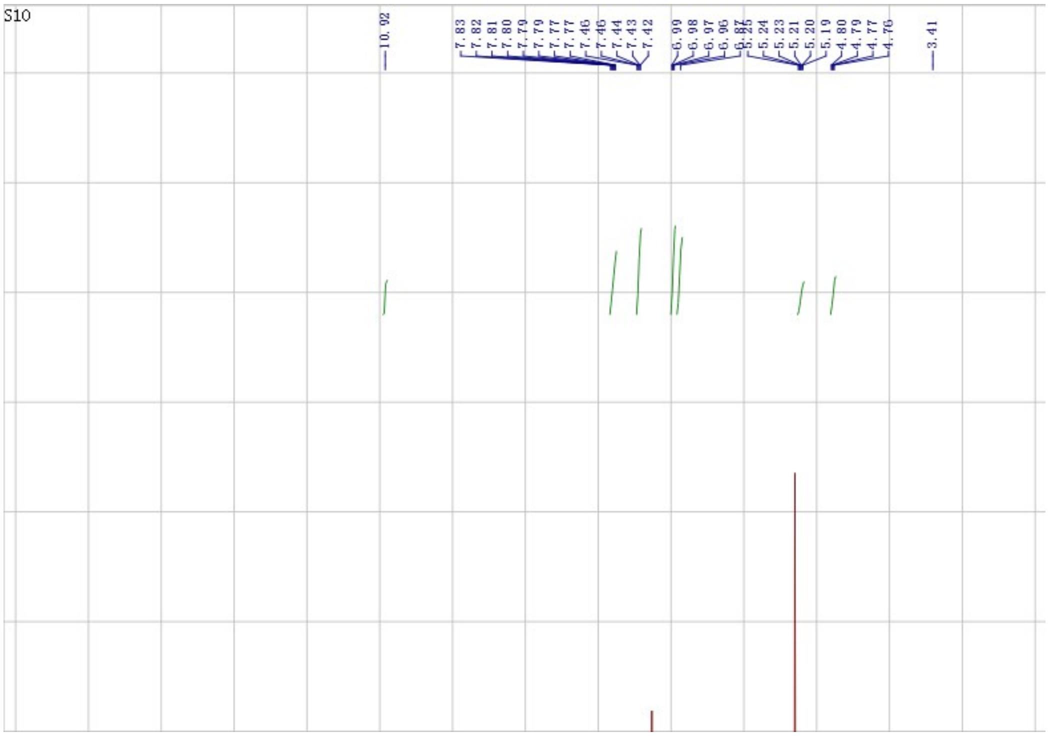

Figure S13. ^{1}H-NMR of compound **7a**

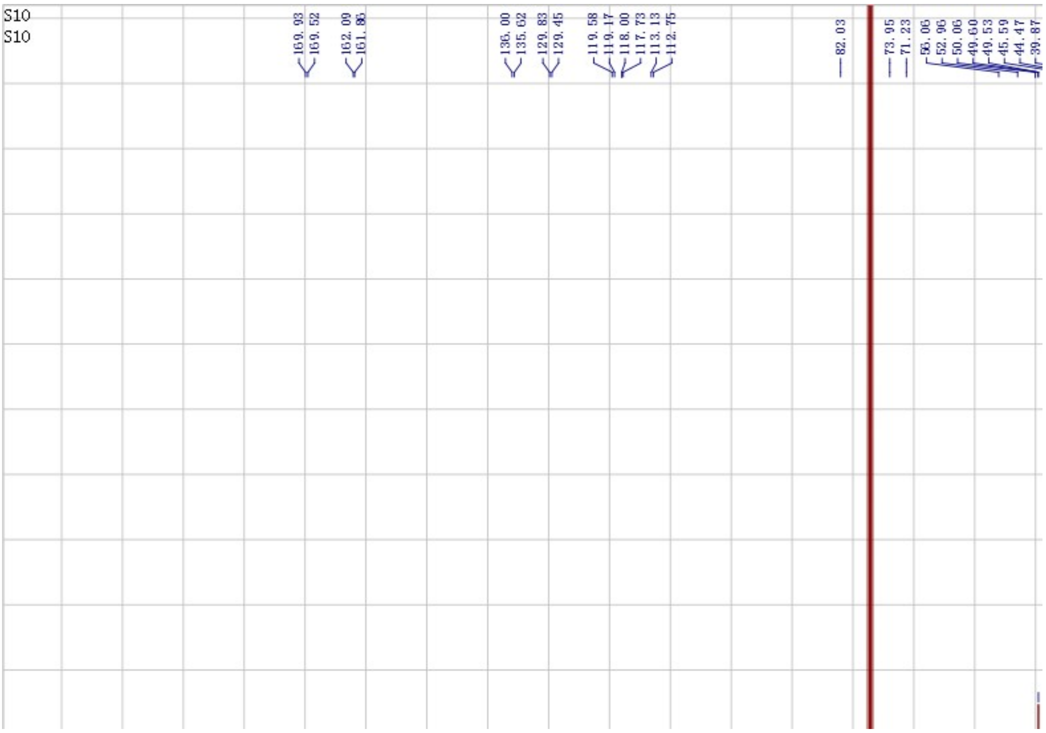

Figure S14. ^{13}C-NMR of compound **7a**

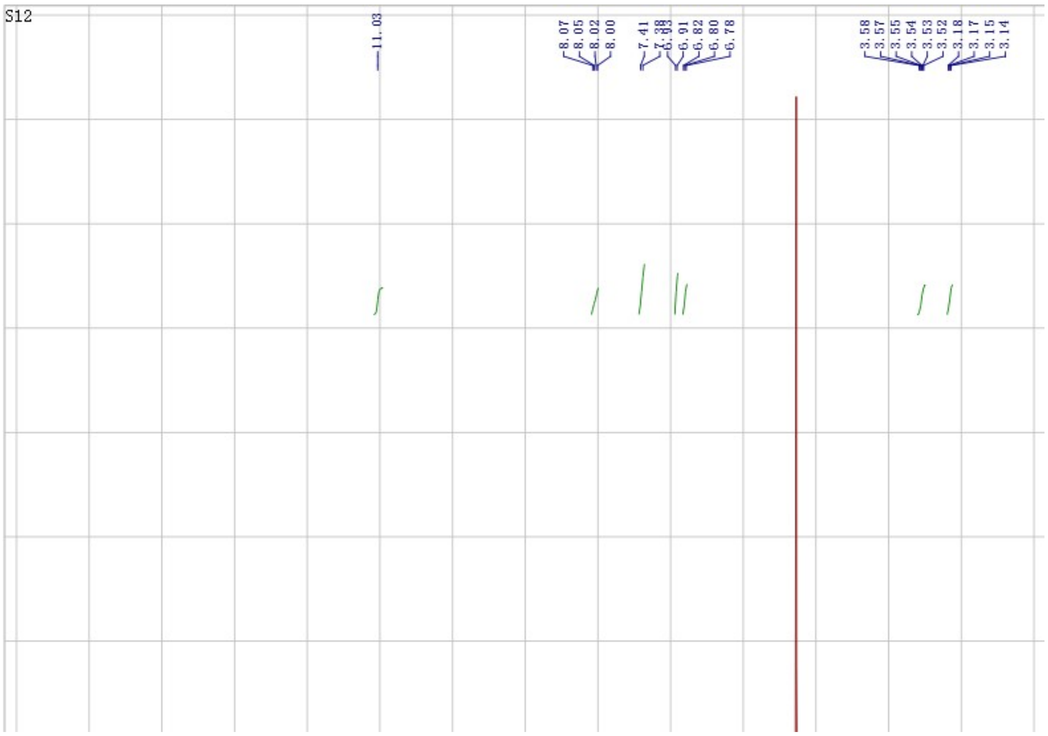

Figure S15. ^{1}H-NMR of compound **8a**

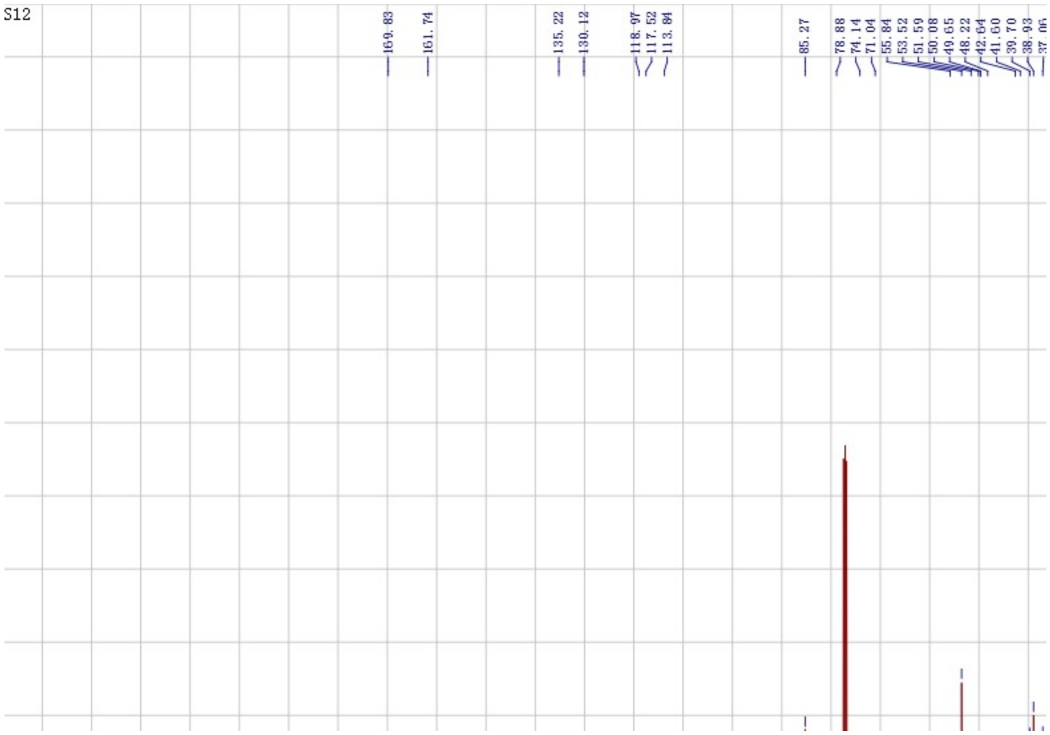

Figure S16. ^{13}C-NMR of compound **8a**

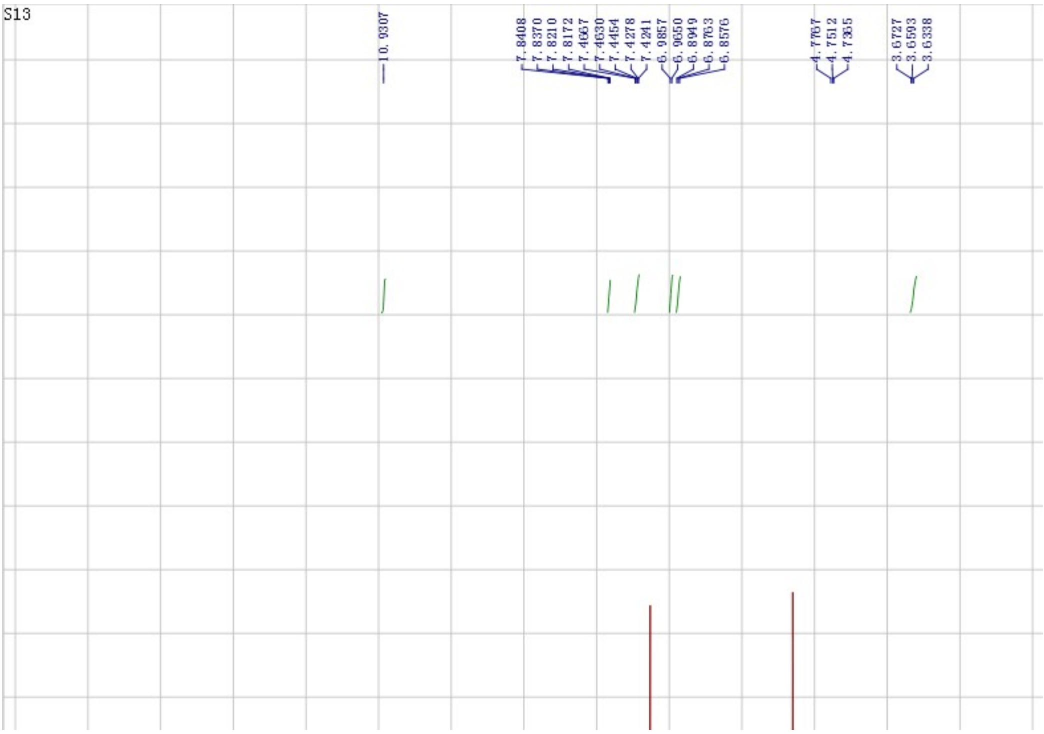

Figure S17. ^{1}H-NMR of compound **9a**

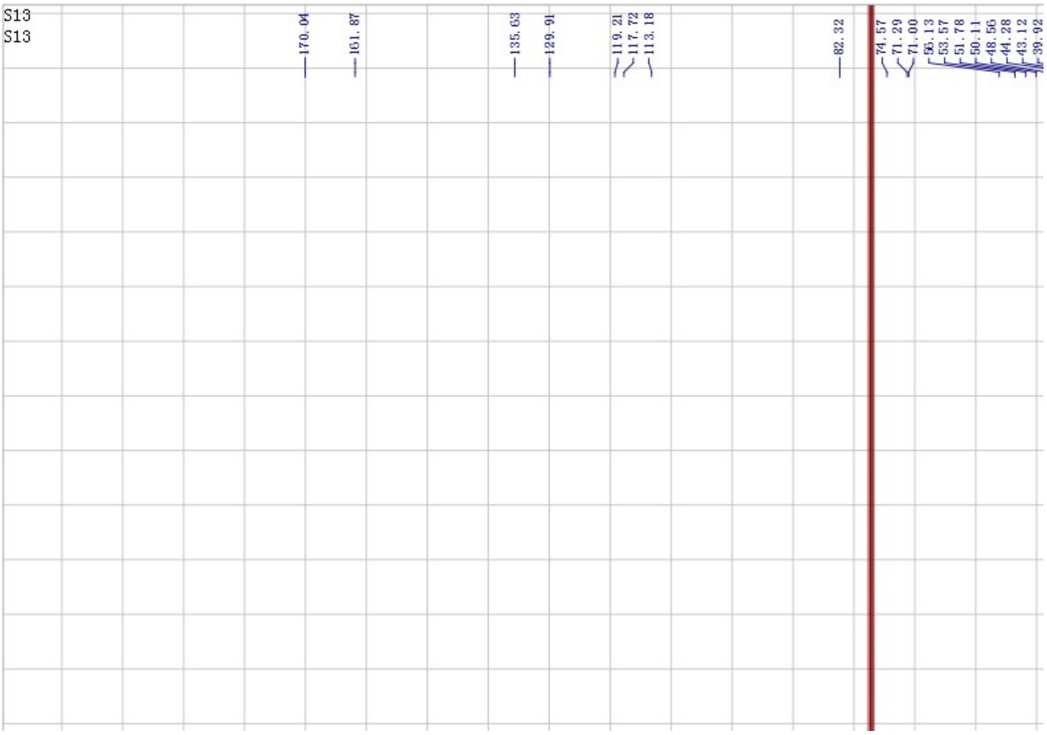

Figure S18. ^{13}C-NMR of compound **9a**

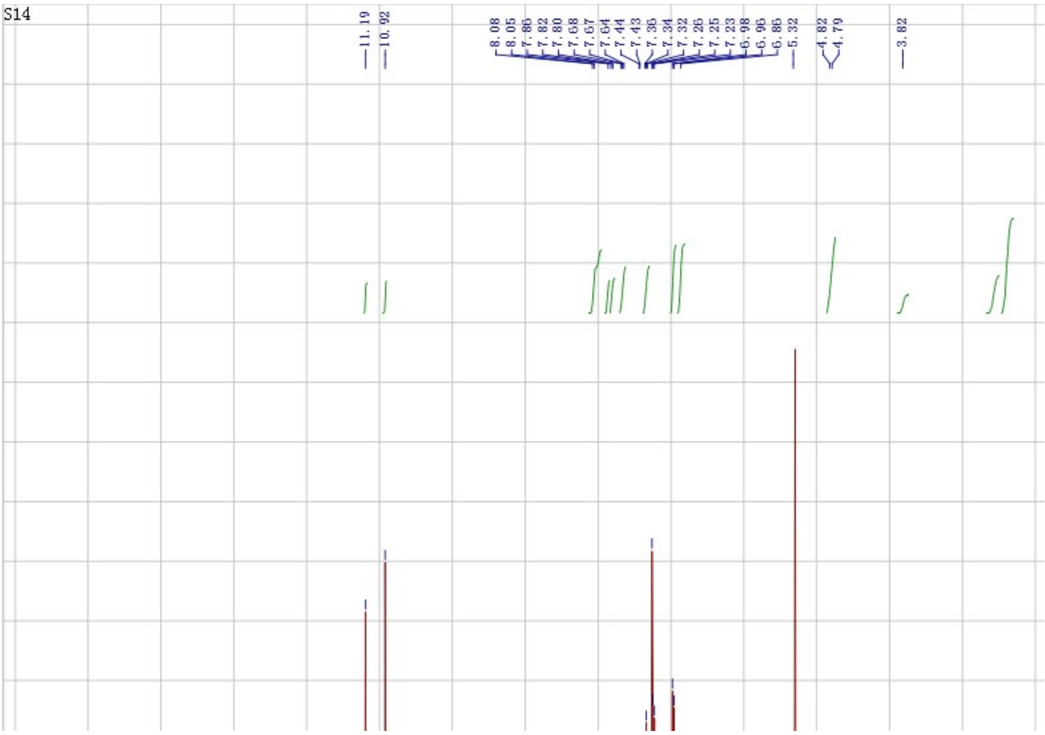

Figure S19. ^{1}H-NMR of compound **10a**

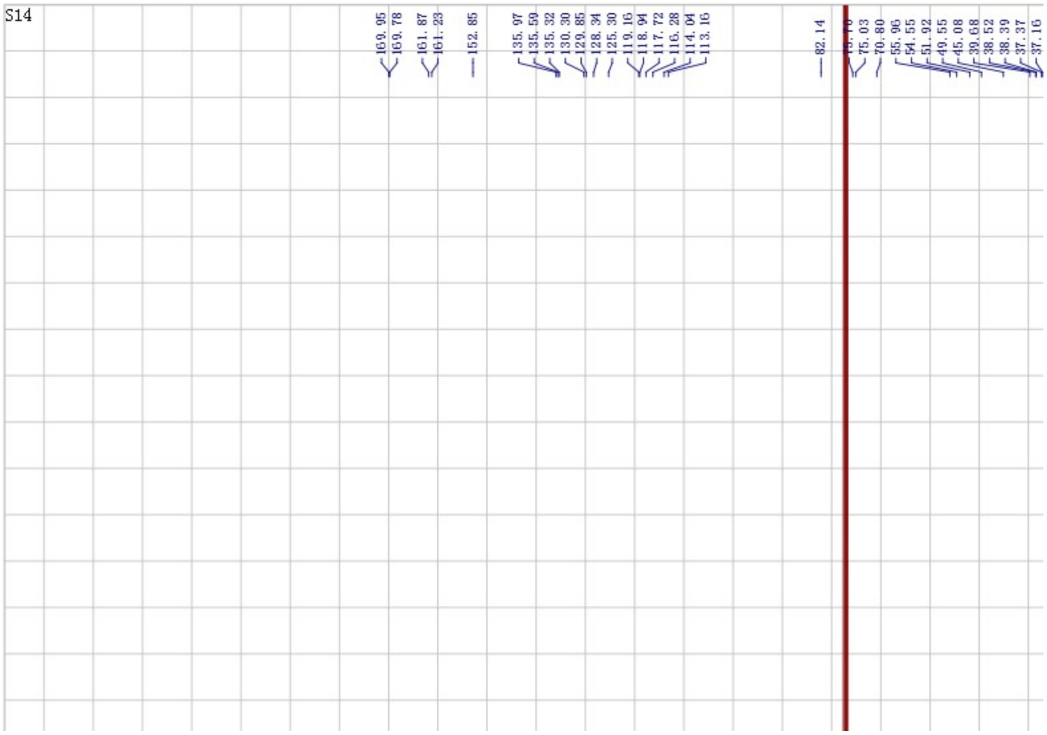

Figure S20. ^{13}C-NMR of compound **10a**

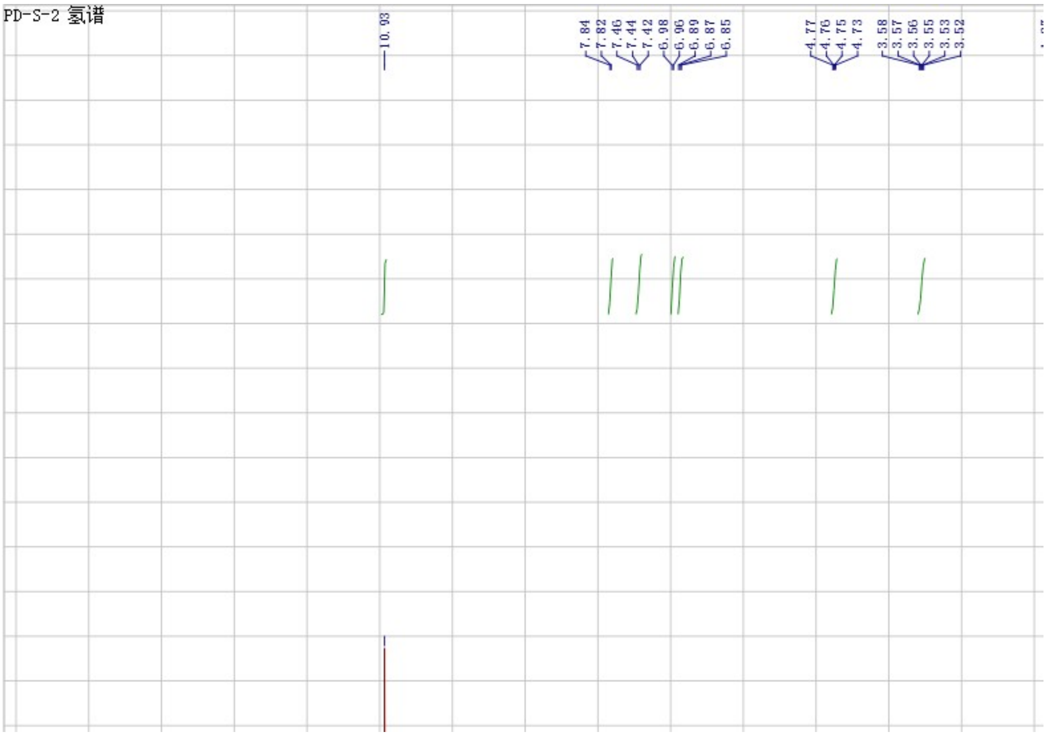

Figure S21. ^{1}H-NMR of compound **11a**

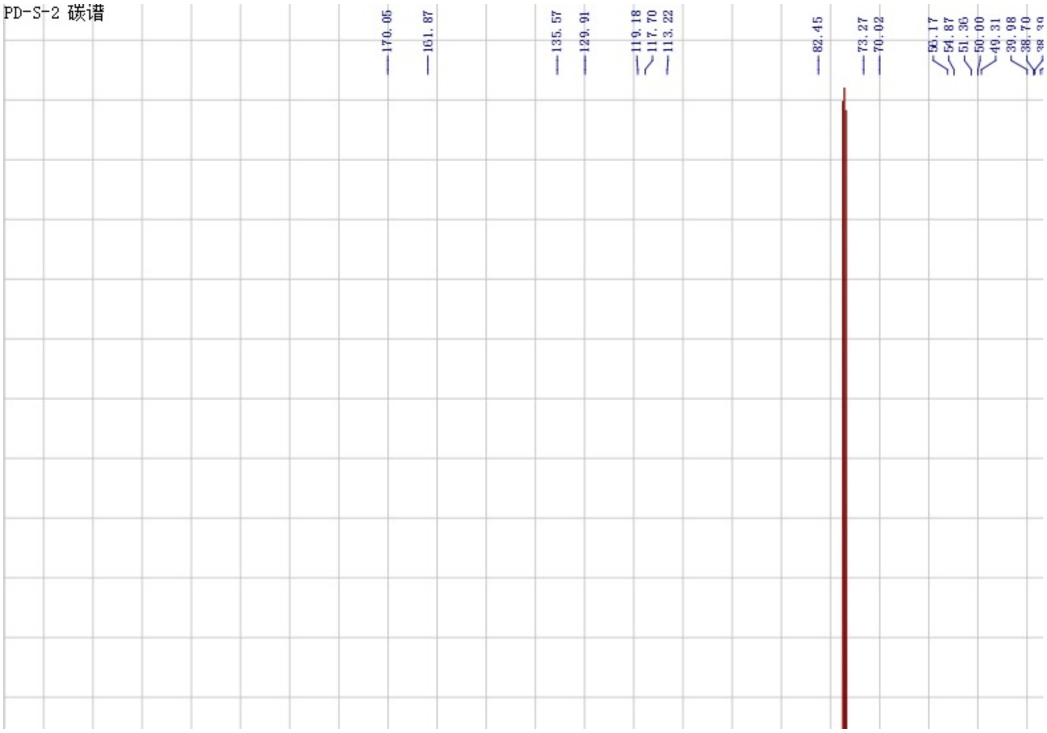

Figure S22. ^{13}C-NMR of compound **11a**

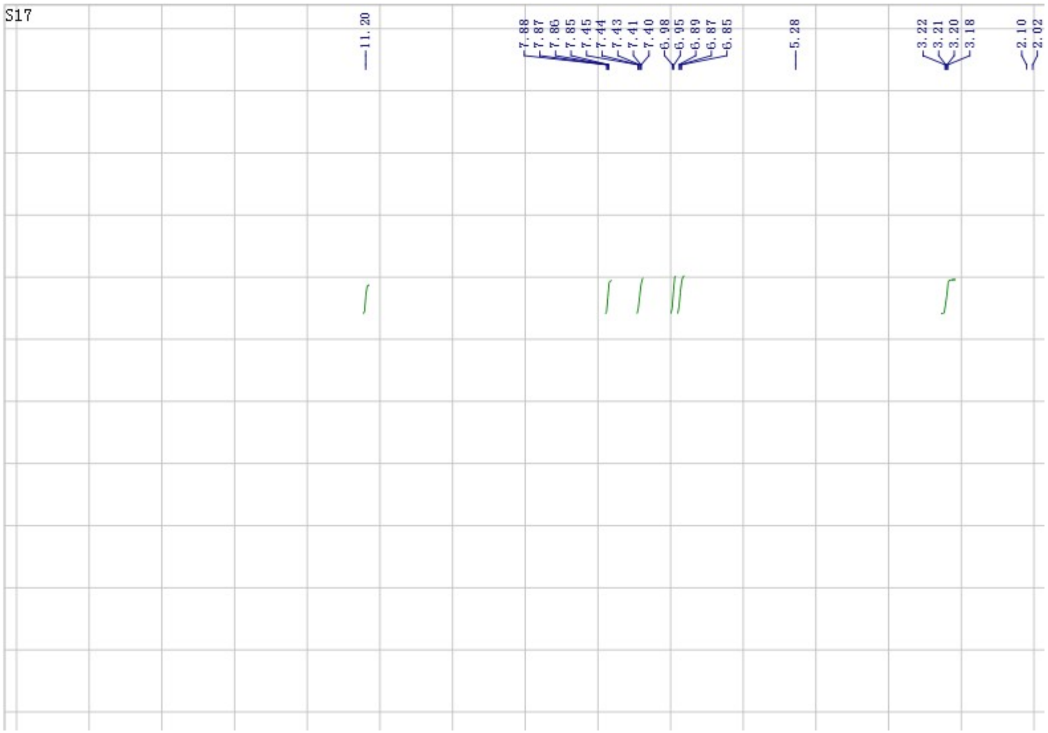

Figure S23. ^{1}H-NMR of compound **12a**

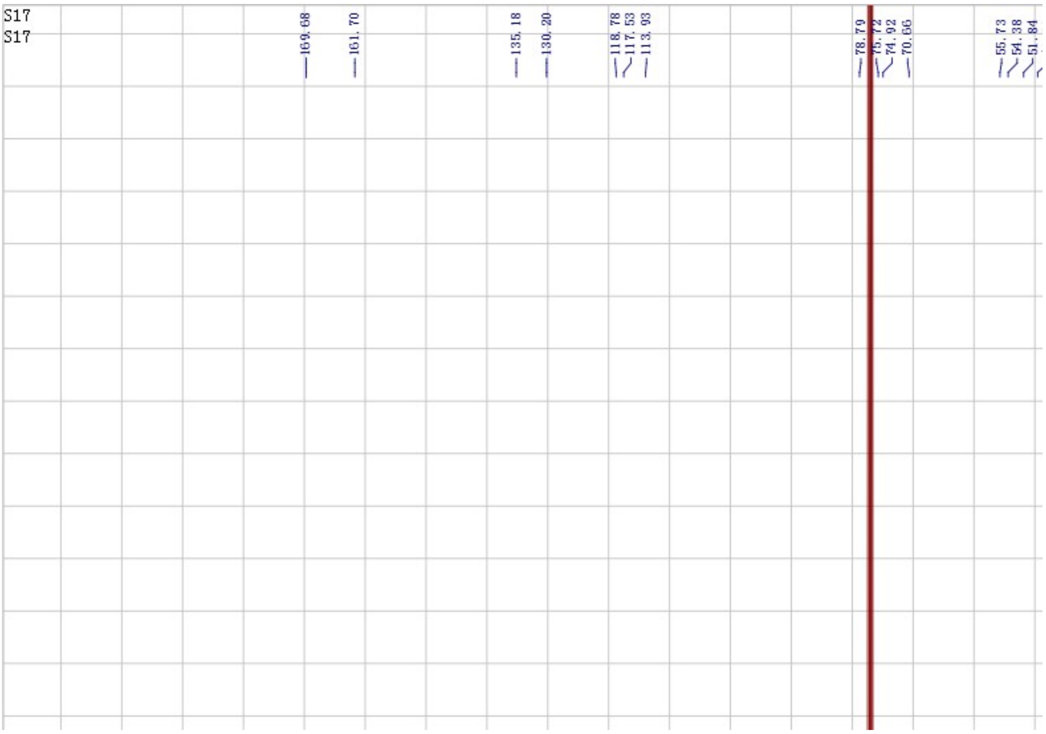

Figure S24. ^{13}C-NMR of compound **12a**

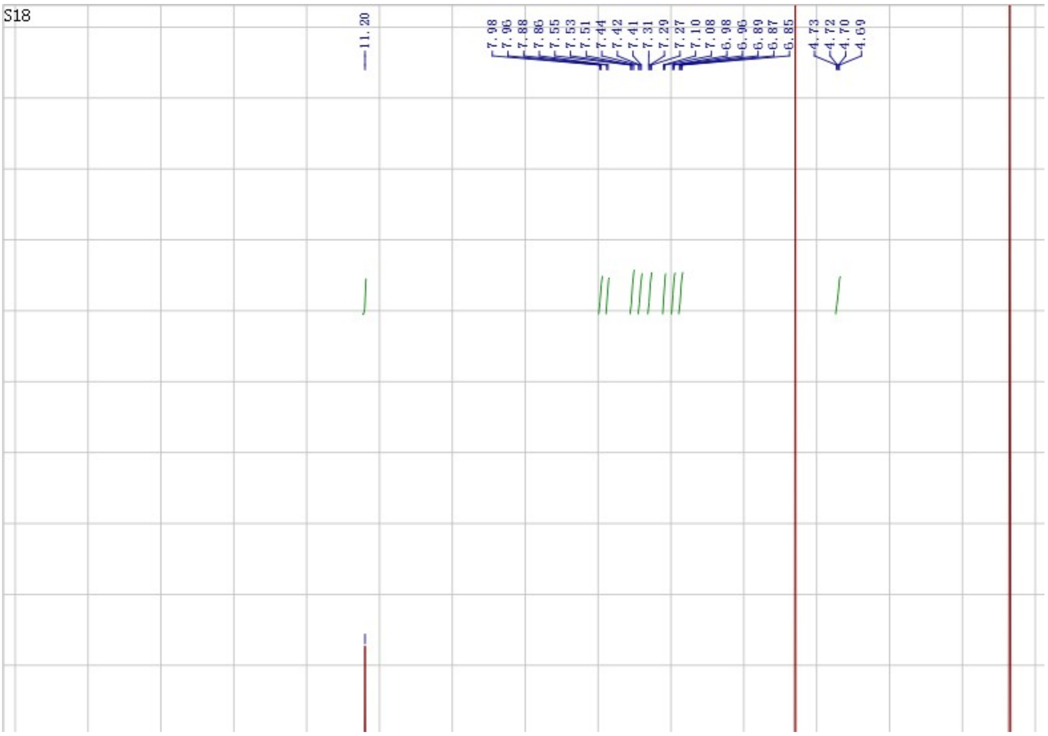

Figure S25. ^{1}H-NMR of compound **1b**

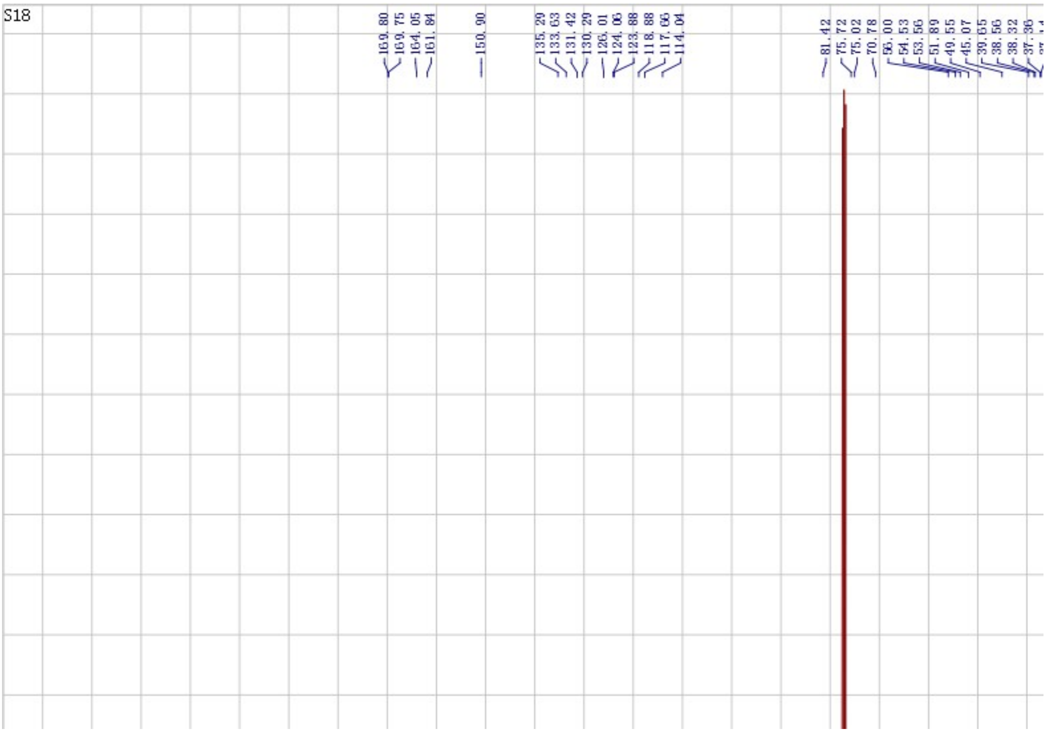

Figure S26. ^{13}C-NMR of compound **1b**

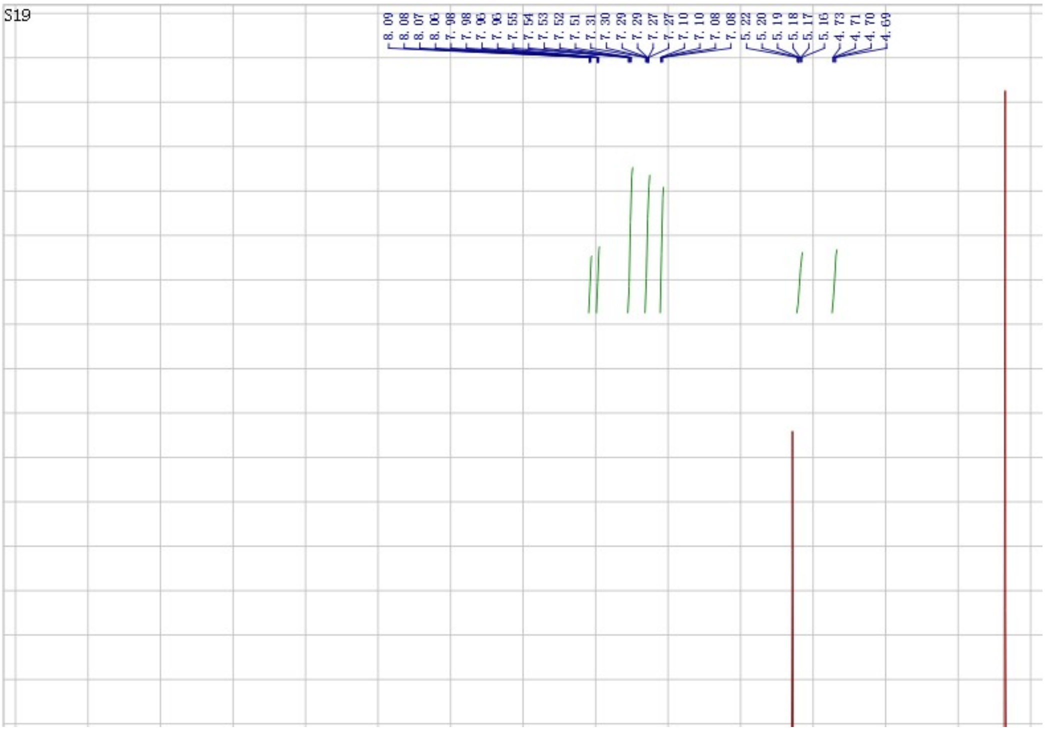

Figure S27. ^{1}H-NMR of compound **2b**

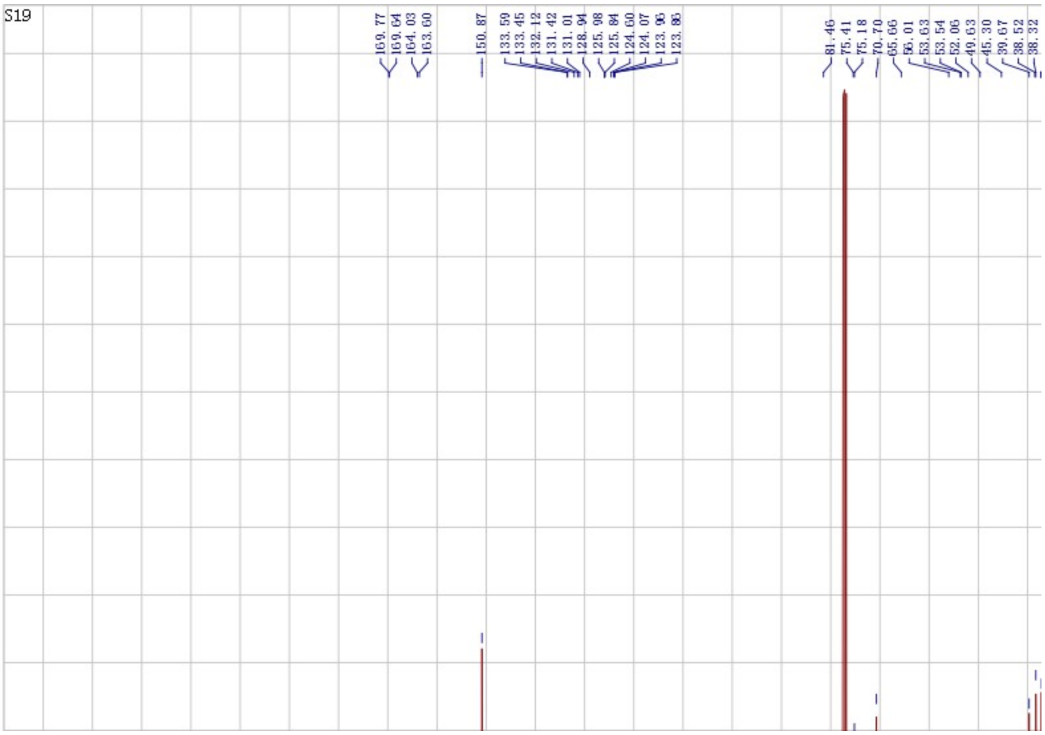

Figure S28. ^{13}C-NMR of compound **2b**

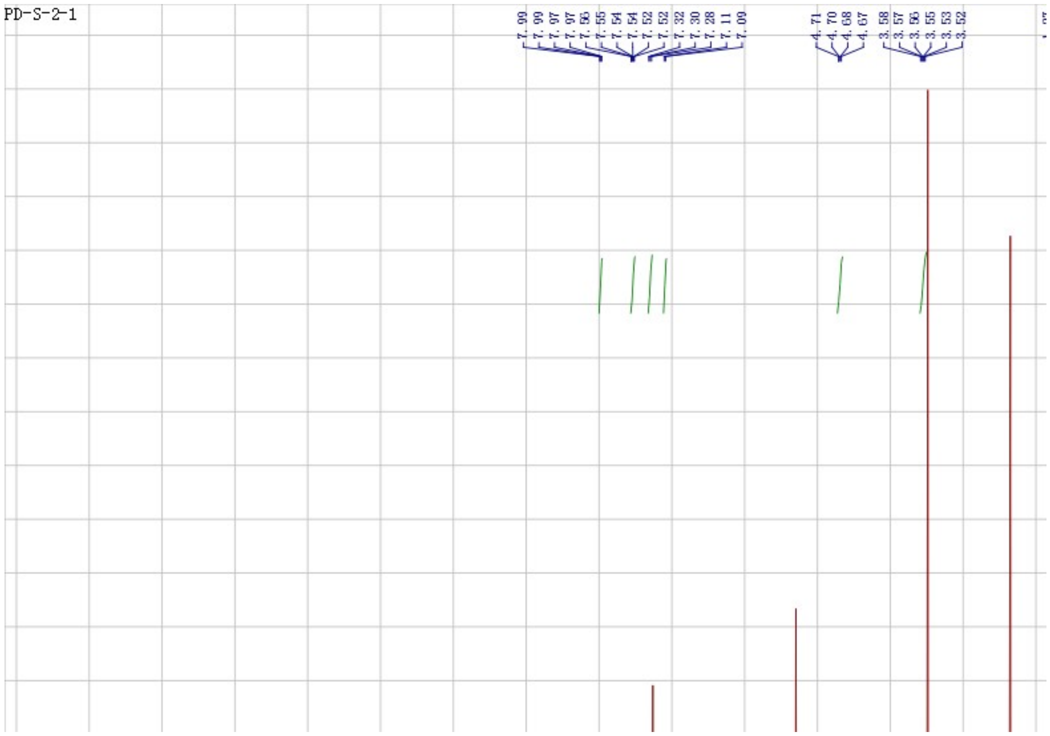

Figure S29. ^{1}H-NMR of compound **3b**

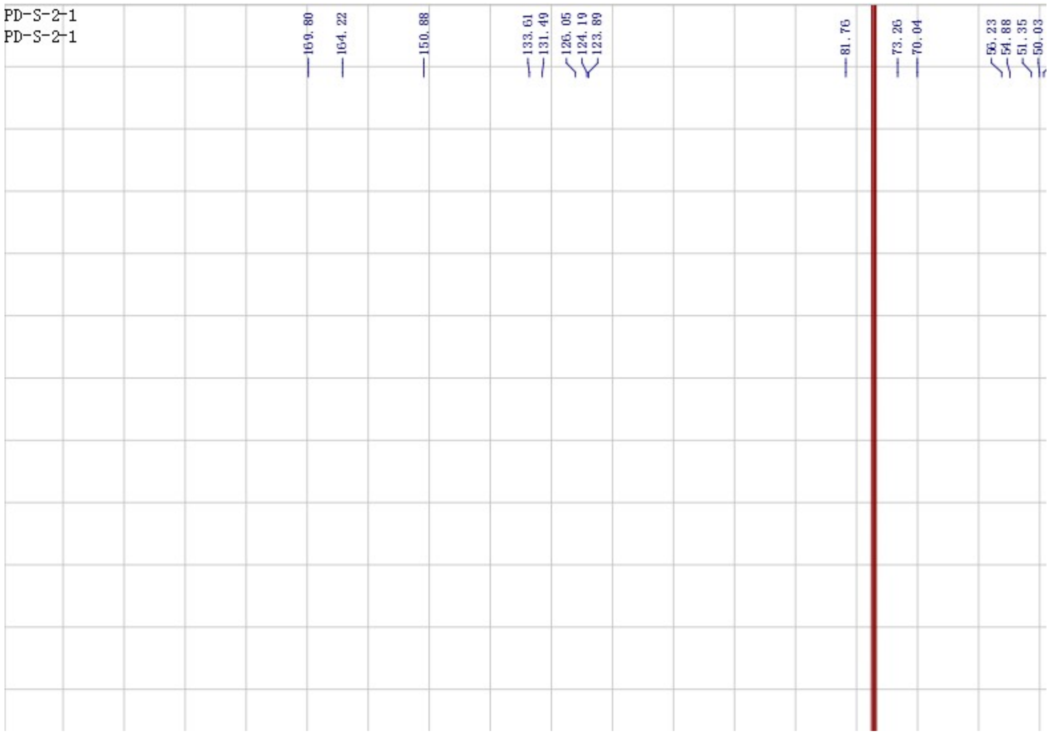

Figure S30. ^{13}C-NMR of compound **3b**

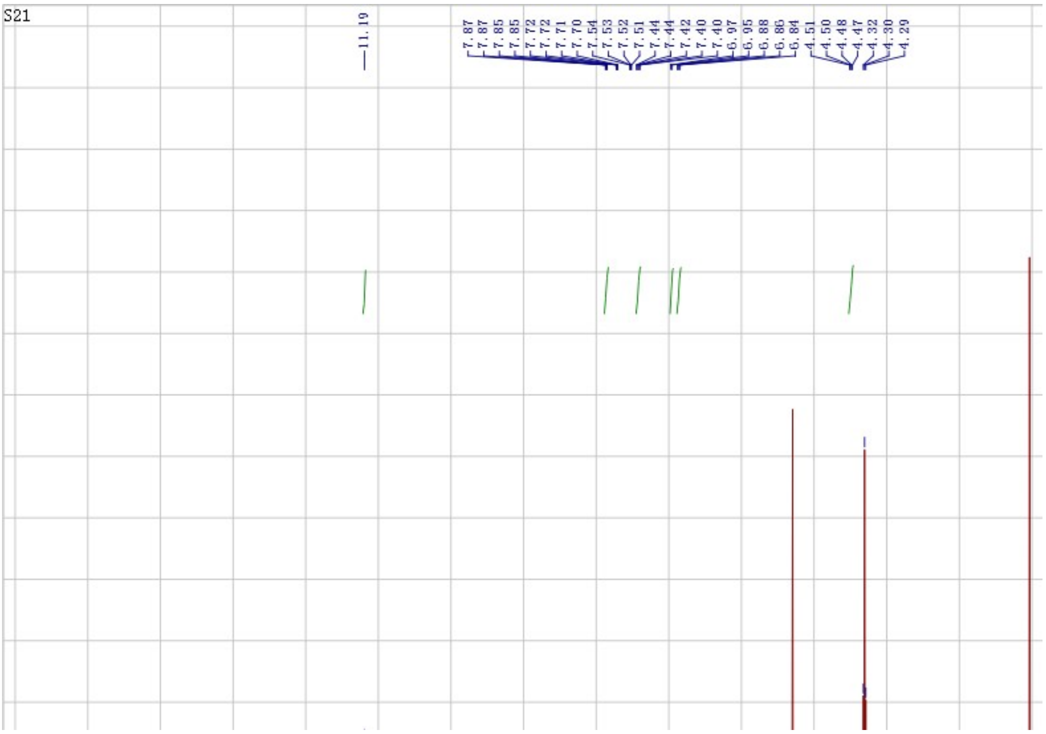

Figure S31. ^{1}H-NMR of compound **4b**

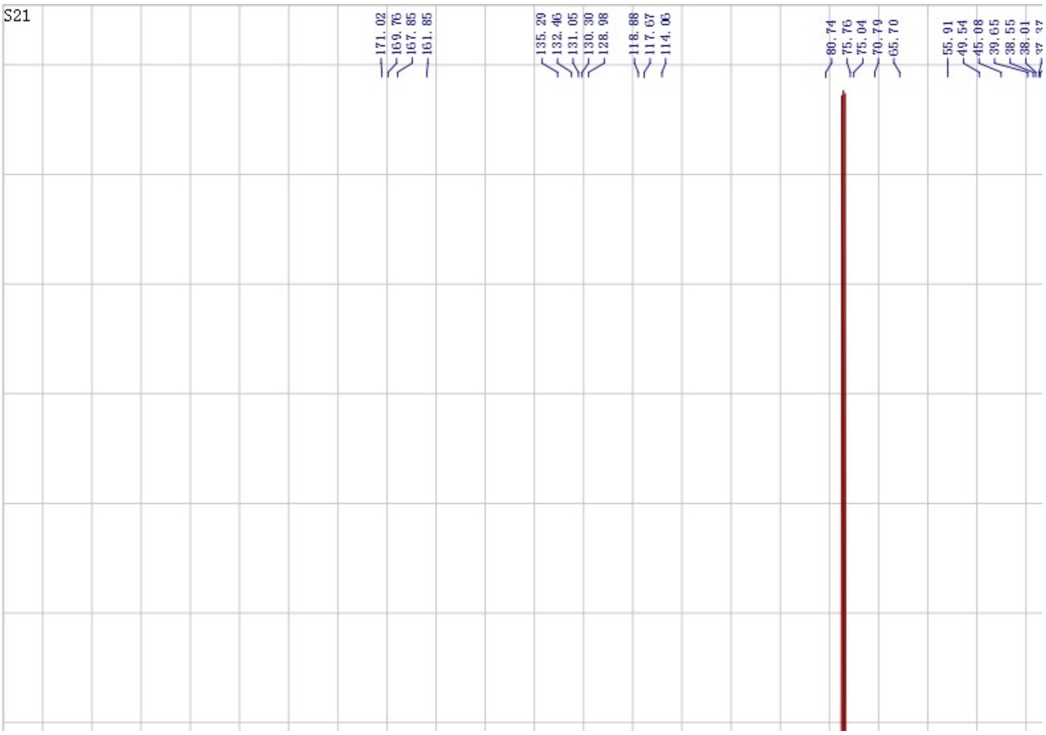

Figure S32. ^{13}C-NMR of compound **4b**

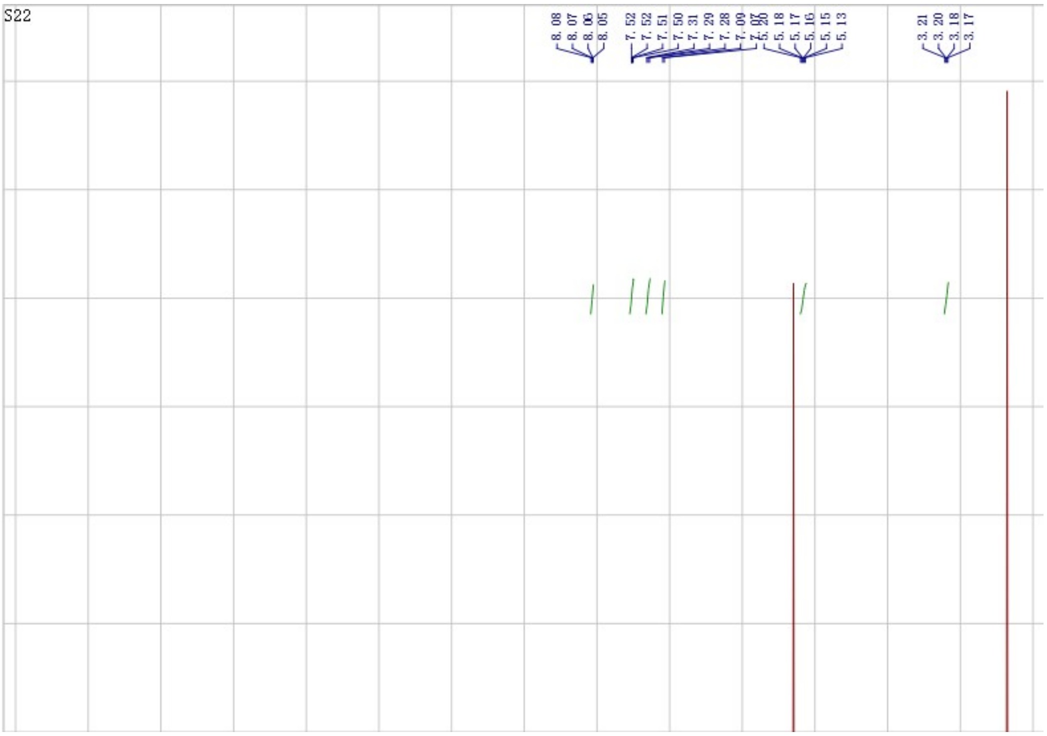

Figure S33. ^{1}H-NMR of compound **5b**

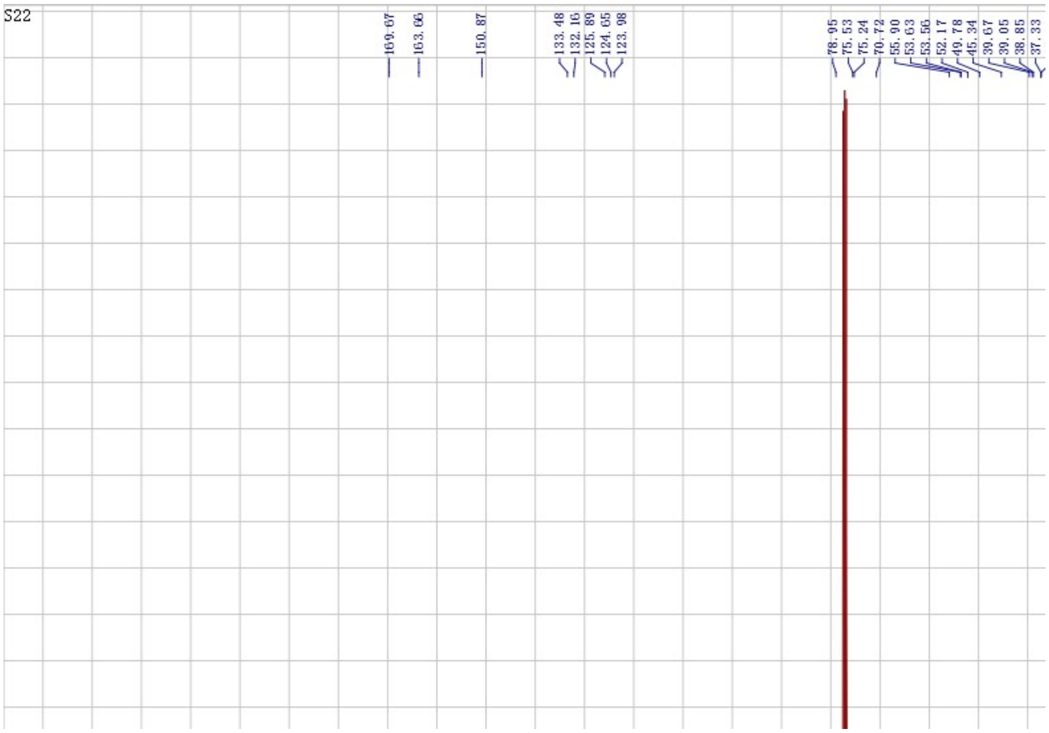

Figure S34. ^{13}C-NMR of compound **5b**

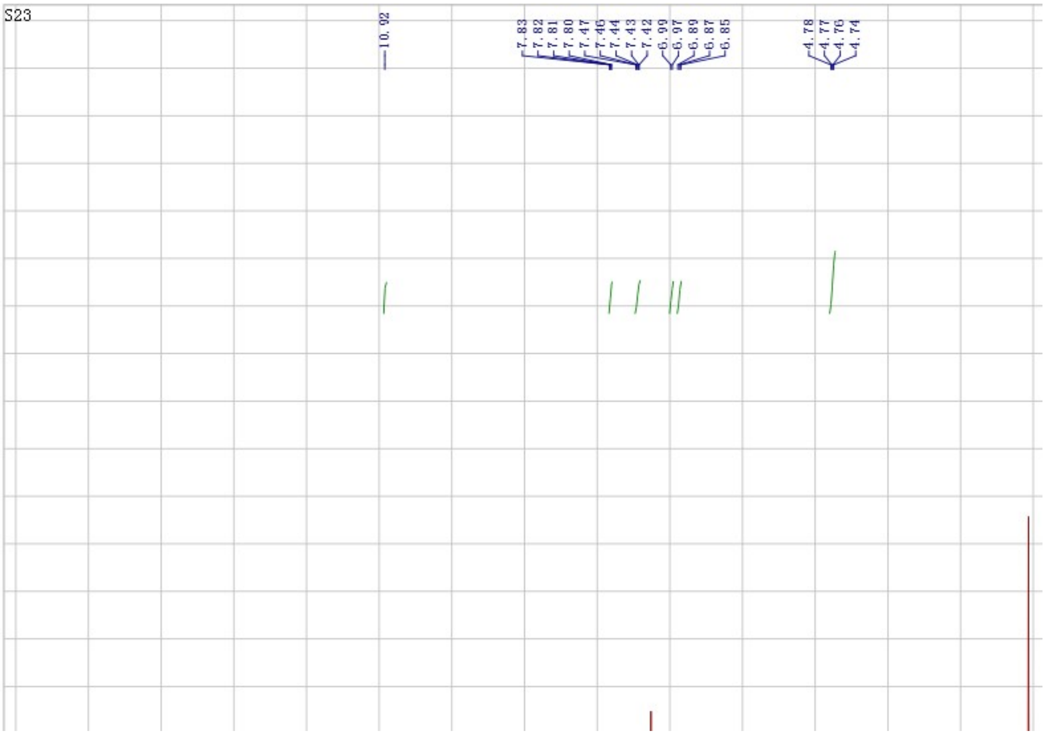

Figure S35. ^{1}H-NMR of compound **6b**

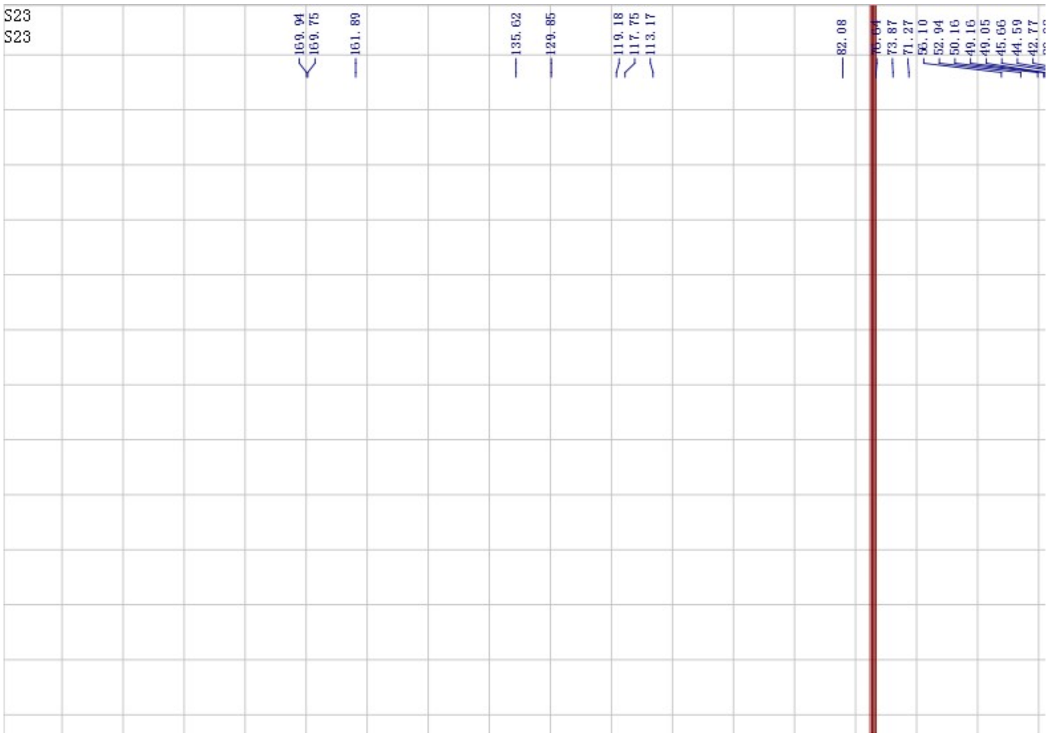

Figure S36. ^{13}C-NMR of compound **6b**

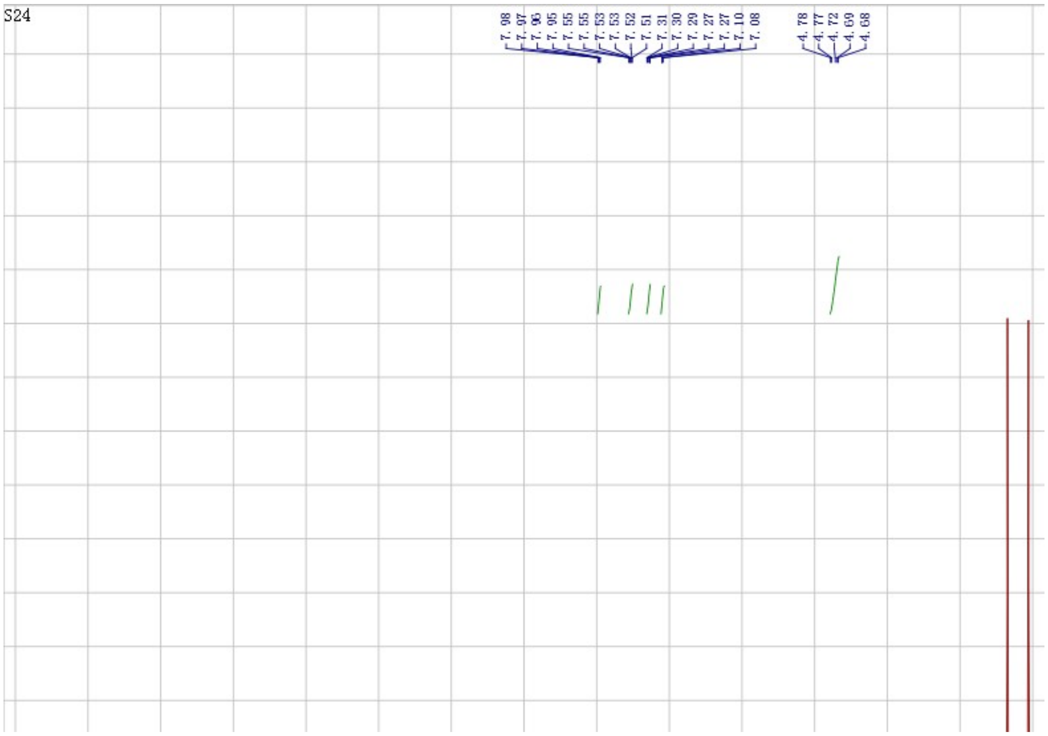

Figure S37. ^{1}H-NMR of compound **7b**

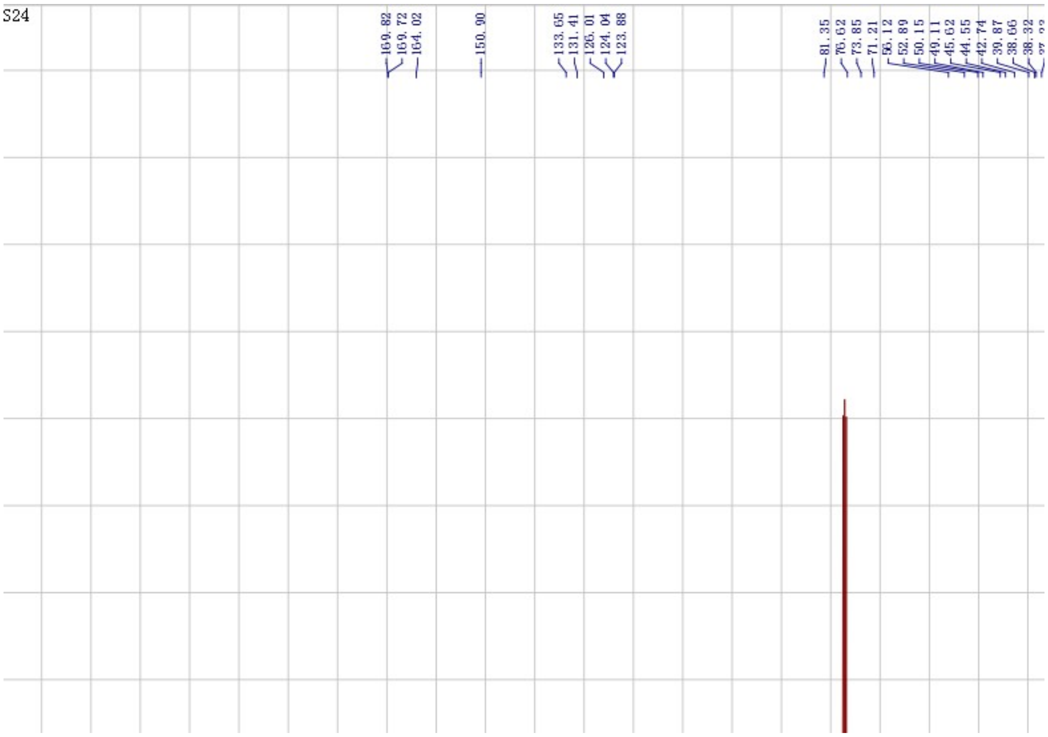

Figure S38. ^{13}C-NMR of compound **7b**

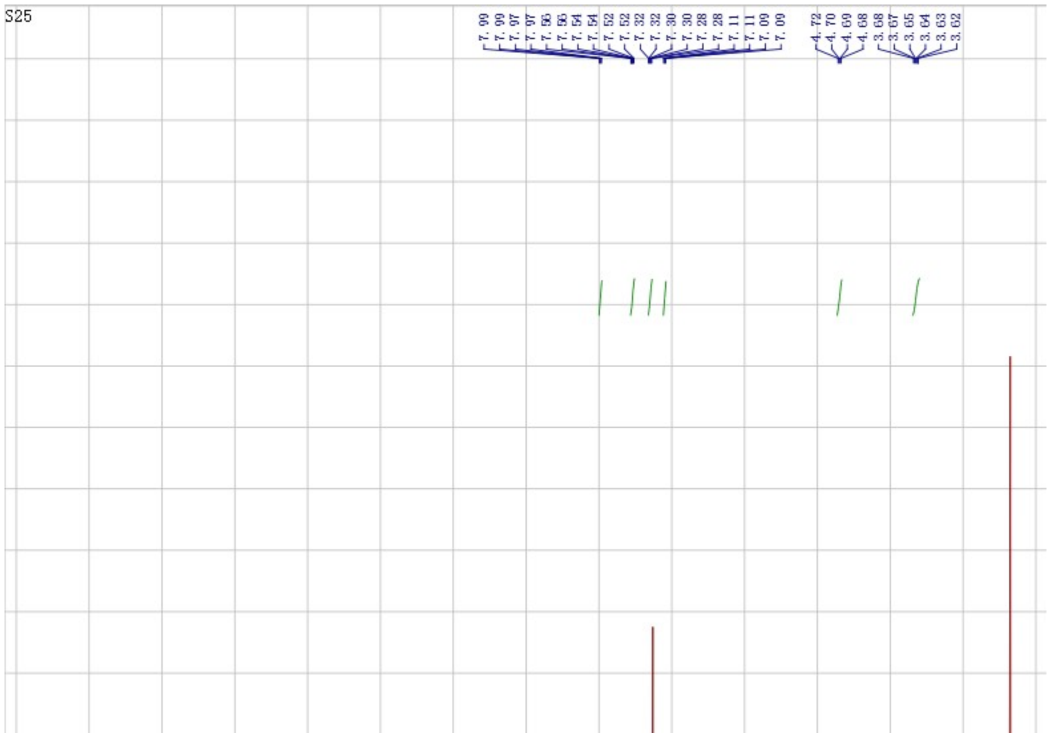

Figure S39. ^{1}H-NMR of compound **8b**

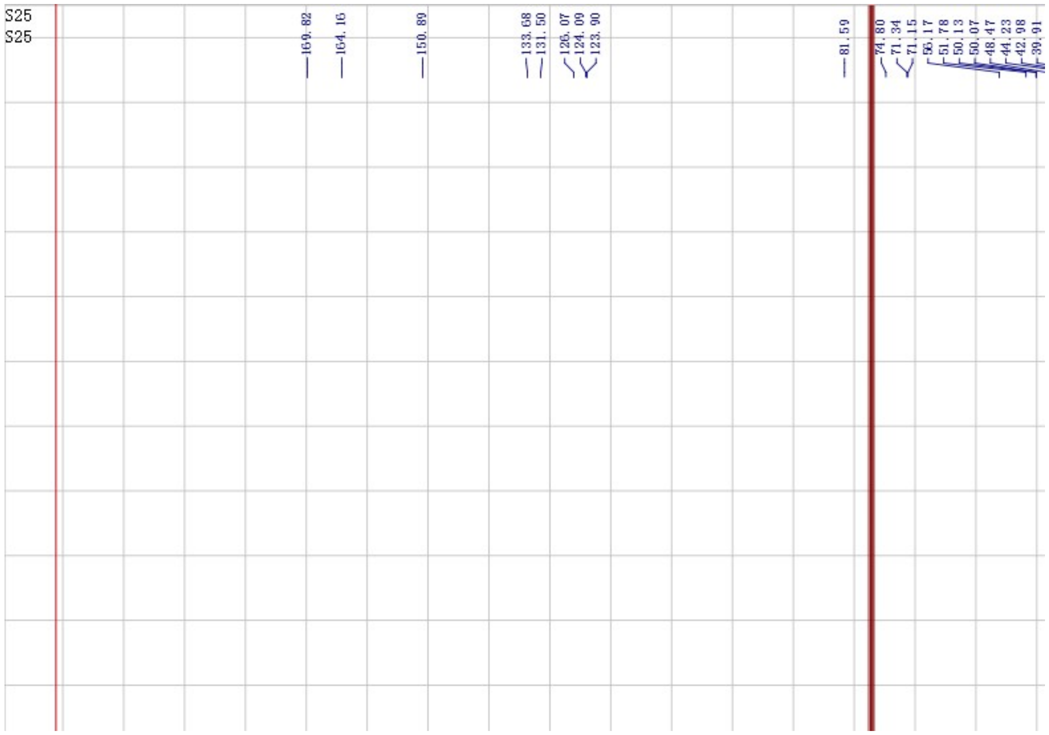

Figure S40. ^{13}C-NMR of compound **8b**
